# Catalogue of fungi in China 2. *Ramaria* from northern China

**DOI:** 10.1080/21501203.2024.2388910

**Published:** 2024-10-28

**Authors:** Ying Li, Ning Mao, Yu-Xing Zhang, Hao-Yu Fu, Li Fan

**Affiliations:** College of Life Science, Capital Normal University, Beijing, China

**Keywords:** Coralloid-fungi, Gomphales, molecular analysis, taxonomy

## Abstract

The species of *Ramaria* are coralloid-fungi with intricately branched and vividly coloured basidiomata. There are many studies on the genus *Ramaria* in China, but molecular phylogenetic analysis is rarely employed. In this study, we performed a multigene phylogenetic analysis to identify *Ramaria* species in Shanxi Province of northern China. Phylogenetic analyses based on four loci, the internal transcribed spacer region of nuclear ribosomal DNA (ITS), the nuclear large subunit ribosomal DNA (nrLSU), ATPase subunit 6 (*atp6*), and mitochondrial small subunit ribosomal DNA (mtSSU), revealed 13 *Ramaria* species from our collections. Combined with morphological examinations, 12 of them were identified as new species, plus a new report to China. The thirteen species were described and illustrated in this paper.

## 1. Introduction

The genus *Ramaria* Fr. ex Bonord. (Gomphaceae, Gomphales) is coralloid-fungi with intricately branched and vividly coloured basidiomata (Marr and Stuntz 1973; Petersen 1981; Humpert et al. 2001). Important diagnostic characteristics include colour, stipe consistency, odour, staining reactions to reagents, presence or absence of clamp connections, spore size, and ornamentation (Corner 1950; Marr and Stuntz 1973; Petersen 1981; Humpert et al. 2001; Exeter et al. 2006). The species in this genus have different lifestyles, including ectomycorrhizal, saprobic, lignicolous, or humicolous (Marr and Stuntz 1973; Exeter et al. 2006; González et al. 2023). They thus play a significant role in forest development and ecological balance. Some species are taken as precious and delicious food, such as *R. botrytis* (Pers.) Ricken, *R. pallidolilacina* P. Zhang and Z.W. Ge, *R. subbotrytis* (Coker) Corner (Corner 1950; Zhang et al. 2005; Liu et al. 2013; Sunil et al. 2013). Molecular analyses revealed that *Ramaria* is a paraphyletic genus (Marr and Stuntz 1973; Humpert et al. 2001; Exeter et al. 2006; Giachini et al. 2010; González et al. 2023). Currently, more than 600 binomials are included worldwide in the Index Fungorum database (www.indexfungorum.org, accessed 28 February 2024).

In China, sixty *Ramaria* species have been reported, but only four species are confirmed by molecular data (Deng 1968; Dai 1979; Mao 2000; Zhang et al. 2005; Dai et al. 2010; Gao and Sun 2017; Chen and Zhang 2019; Miyauchi et al. 2020; Wang et al. 2022). Shanxi Province is located in the hinterland of northern China, ranging from 34°34’N to 40°44’N and 110°14’E to 114°33’E, with a temperate continental climate. It is mountainous, most areas of which have an altitude of more than 1,000 metres. From 2018 to 2023, a large collection of *Ramaria* specimens was obtained in this province. Twelve new species and a new report to China were recognised from these collections based on morphological examinations and molecular phylogenetic analyses. In the present study, these species were described and illustrated.

## 2. Materials and methods

### 2.1. Morphological studies

Specimens were collected in Shanxi Province, northern China, and photographed in the field, then dried in a fruit dryer at 40 − 45 °C and deposited in BJTC (Herbarium Biology Department, Capital Normal University, Beijing, China) and the HSA (Herbarium Institute of Edible Fungi, Shanxi Academy of Agricultural Science, Taiyuan, China). Macroscopic characteristics were based on fresh specimens and photographs. Standardised colour values matching the colours of the description were taken from ColorHexa (http://www.colorhexa.com). Microscopic characteristics were conducted on sections from dried material and rehydrated in 3% KOH (Henderson et al. 1970). Moreover, ferric sulphate (10% aqueous solution) was used to test the macrochemical reaction (positive: turning some shade of green; negative: no shade of green or other colour change); Melzer’s reagent was used to test the iodine reaction (positive: amyloid; negative: inamyloid); Cotton blue was used to test the cyanophilous reaction (positive: cyanophilic; negative: non cyanophilic) (Kotlaba and Pouzar 1964; Henderson et al. 1970; Dring 1971; Exeter et al. 2006; González et al. 2023). For each specimen, at least 30 spores and 15 basidia were measured. Dimensions of basidiospores were given using the following format “(a-)b-c(−d)”, where the range “b-c” represents at least 90% of the measured values, and “a” and “d” are the most extreme values. “Q” refers to the length/width ratio in the profile view of basidiospores, and Q_m_ refers to the average Q of all basidiospores ± sample standard deviation. For scanning electron microscopy (SEM), basidiospores were scraped from dried specimens onto doubled-sided tape that was mounted directly on an SEM stub, coated with platinum-palladium film using an ion-sputter coater (Hitachi E-1010; Tokyo, Japan), and examined and photographed with a Hitachi S-4800 SEM.

### 2.2. DNA extraction, PCR amplification, and DNA sequencing

Four DNA fragments, ITS, nrLSU, *atp6*, and mtSSU, were obtained in this study. A small amount of dried basidiocarp was crushed by shaking for 45 s at 30 Hz 2–4 times (Mixer Mill MM301, Retsch, Haan, Germany) in a 1.5 mL tube together with a 3-mm-diam tungsten carbide ball and total genomic DNA was extracted using the modified CTAB method (Gardes and Bruns 1993). The primer pairs of ITS1-F/ITS4 for ITS (internal transcribed spacer rDNA region) (White et al. 1990; Gardes and Bruns 1993), primers LROR/LR3 for nrLSU (nuclear large subunit ribosomal DNA) (Vilgalys and Hester 1990), primers MS1/MS2 for mtSSU (mt small subunit rDNA) (White et al. 1990; Liu et al. 2023), and primers ATP6-3/ATP6-4 for *atp6* (ATPase subunit 6) (Giachini et al. 2010) were used to amplify and sequence the four loci mentioned above. Polymerase chain reactions (PCR) were performed in a 25 µL reaction containing 2 µL and 9.2–12.3 ng/µL DNA template, 1 µL primer (10 µmol/L) each, 12.5 µL of 2 × Master Mix [Tiangen Biotech (Beijing) Co.], 8.5 µL ddH_2_O. Amplification reactions were performed as follows: for the ITS and nrLSU regions: initial denaturation at 94 °C for 1 min, followed by 34 cycles at 94 °C for 1 min, 50 °C for 30 s, 72 °C for 45 s, and a final extension 72 °C for 3 min; for the mtSSU gene: initial denaturation at 94 °C for 4 min, followed by 35 cycles at 94 °C for 30 s, 45 °C for 45 s, 72 °C for 1 min, and a final extension at 72 °C for 10 min; for the *atp6* gene: initial denaturation at 94 °C for 3 min, followed by 35 cycles at 94 °C for 30 s, 43 °C for 45 s, 72 °C for 1 min, and a final extension at 72 °C for 10 min. The PCR products were sent to Sangon Biotech (Shanghai) Co., Ltd. for purification, sequencing, and editing.

### 2.3. Phylogenetic analyses

Validated sequences newly sequenced were deposited in GenBank database. Other sequences of *Ramaria* and related species were retrieved from GenBank database by BLASTn search, or selected from those used by Giachini et al. (2010), Martín et al. (2020), and Xu et al. (2022). The accession numbers of all sequences used are provided in Tables 1 and 2. Two datasets, a combined dataset (ITS-nrLSU-*atp6*-mtSSU) and a single gene dataset (ITS), were used to investigate the phylogenetic positions of our collections in the genus *Ramaria*. The reason for employing ITS dataset was in order to include as many species as possible in phylogenetic analysis, as some *Ramaria* species only have ITS sequences available. *Phallus impudicus* L. and *Mutinus elegans* (Mont.) E. Fisch. were selected as outgroups based on the previous studies (Giachini et al. 2010; Xu et al. 2022). The sequences of each marker were independently aligned in MAFFT v.7.110 (Katoh and Standley 2013) under default parameters. Poorly aligned sites were identified by Gblocks v.0.91b (http://www.phylogeny.fr/one_task.cgi?task_type=gblocks; using default options except ALLOWED GAP POSITIONS 5 half) with default parameters. All identified ambiguous sites were excluded before the analyses. Maximum Likelihood (ML) and Bayesian Inference (BI) analyses were conducted on the resulting datasets.

**Table 1.** Specimens used in molecular phylogenetic studies and their GenBank and UNITE accession numbers.

Taxon	Voucher ID	Country	ITS
*Mutinus elegans*	ME.BST	USA	OQ694424
*Phallus dongsun*	GDGM 75402	China	NR_171851
*Ramaria abetonensis*	MCVE 28638	Italy	KT357472
*Ramaria abetonensis*	AH 48006	Spain	MH322661
*Ramaria acris*	Strain UT-36052-T	USA	OM238175
*Ramaria acrisiccescens*	OSC 112057	USA	KY354738
*Ramaria acrisiccescens*	RLE2005-100	USA	KY986440
*Ramaria acrisiccescens*	OSC 87690	USA	AY102857
*Ramaria acrisiccescens*	OSC 87692	USA	AY102858
*Ramaria admiratia*	TFB14450	USA	KJ416133
*Ramaria admiratia*	TENN 69114	USA	NR_137862
*Ramaria albidoflava*	AMB 18606	France	MT452502
*Ramaria albidoflava*	ZT Myc 55621	Spain	MK493033
*Ramaria amyloidea*	OSC 96384	USA	EU697257
*Ramaria amyloidea*	OSC 66957	USA	EU697258
*Ramaria anziana*	TENN 43401	New Zealand	MF564295
***Ramaria apicaliochracea***	**BJTC FM2738**	**China**	**PP537936**
***Ramaria apicaliochracea***	**BJTC FM1078**	**China**	**PP537935**
*Ramaria apiculata*	MA-Fungi 48064	Spain	AJ408385
*Ramaria apiculata*	HC-PNNT-263	Mexico	KT307869
*Ramaria araiospora*	OSC 104945	USA	EU669243
*Ramaria araiospora*	OSC 134758	USA	KY354758
*Ramaria atractospora*	AMB 18598	Spain	NR_174823
*Ramaria aurantiisiccescens*	CB 08113	Mexico	KT307875
*Ramaria aurantiisiccescens*	HC-PNNT-250	Mexico	KT307873
*Ramaria aurantiisiccescens*	OSC 97400	USA	KP658121
*Ramaria aurantiisiccescens*	OSC 112055	USA	KY354744
*Ramaria aurea*	MA-Fungi 48120	Germany	AJ408387
*Ramaria barenthalensis*	130822MFBPC356	China	MW554136
*Ramaria barenthalensis*	AMB 17386	Spain	MK493039
***Ramaria barenthalensis***	**BJTC FM2750**	**China**	**PP537924**
*Ramaria bataillei*	MA-Fungi 48075	Spain	AF441082
*Ramaria boreimaxima*	H, Kytovuori 96-525	Finland	KJ464996
*Ramaria botrytis*	SNF 213	USA	AF377055
*Ramaria botrytis*	DGUM 29001	South Korea	AY588247
*Ramaria botrytis*	DARD-112	India	KJ184344
*Ramaria botrytis*	MA-Fungi 47951	Spain	AJ292294
*Ramaria botrytis*	AMB 18201	Italy	KY626151
*Ramaria botrytis*	NIFoS1018	Korea	MF421105
*Ramaria brunneolilacina*	AMB 18584	Spain	NR_174822
*Ramaria brunneolilacina*	AMB 18591	Spain	MT055972
***Ramaria cadmioaurantiaca***	**BJTC HSA288**	**China**	**PP537922**
***Ramaria cadmioaurantiaca***	**BJTC HSA280**	**China**	**PP537921**
***Ramaria cadmioaurantiaca***	**BJTC FM2242**	**China**	**PP537920**
*Ramaria calvodistalis*	TENN 69095	USA	KJ416132
*Ramaria canobrunnea*	ZT Myc 54981	Spain	MT055899
*Ramaria cartilaginea*	WTU 43208	USA	KY986436
*Ramaria caulifloriformis*	BEP01 (TENN)	USA	MF755270
*Ramaria cedretorum*	MA-Fungi 48074	Spain	AJ408353
*Ramaria cedretorum*	ZT Myc, Schild 1902	Italy	AJ408392
*Ramaria celerivirescens*	Isolate 137.5	USA	DQ365647
*Ramaria celerivirescens*	OSC 1064227	USA	EU525995
*Ramaria cistophila*	AH 47781	Spain	MF564292
*Ramaria cistophila*	AH 47765	Spain	MF564293
*Ramaria claviramulata*	WTU-F-043055	USA	KX574472
*Ramaria claviramulata*	OSC 1064080	USA	EU525991
*Ramaria comitis*	MA-Fungi 47970	Spain	AF442095
*Ramaria comitis*	AH 48341	Portugal	MH322667
***Ramaria conferta***	**BJTC HSA258**	**China**	**PP537937**
*Ramaria conjunctipes*	OSC 96488	USA	EU846302
*Ramaria conjunctipes*	OSC 119381	USA	EU846304
*Ramaria conjunctipes*	OSC 105346	USA	EU846301
*Ramaria conjunctipes*	OSC 110613	USA	KC346861
*Ramaria coulterae*	TENN 45771	USA	KX574462
*Ramaria coulterae*	OSC 70059	USA	KY354736
*Ramaria cyaneigranosa*	OSC 140651	USA	JX310397
*Ramaria cyaneigranosa*	WTU-F-043056	USA	KX574465
***Ramaria cyanophila***	**BJTC FM2557**	**China**	**PP537933**
***Ramaria cyanophila***	**BJTC HSA260**	**China**	**PP537931**
***Ramaria cyanophila***	**BJTC HSA261**	**China**	**PP537932**
*Ramaria cystidiophora*	UBC F15182	Canada	DQ384590
*Ramaria cystidiophora*	UBC OGTR0419s	Canada	EU597077
*Ramaria dendrophora*	GM19094	Patagonia	OP177715
*Ramaria dendrophora*	GM20020	Patagonia	OP177716
*Ramaria edwinii*	ZT Myc 54975	Spain	MK493034
*Ramaria fagetorum*	MA-Fungi 48117	Spain	AJ408362
*Ramaria fagetorum*	MA-Fungi 48118	Spain	AJ408363
*Ramaria fennica*	MA-Fungi 47996	Spain	AJ408352
*Ramaria flava*	MA-Fungi 48072	Spain	AJ408367
*Ramaria flava*	ZT Myc 55613	Germany	KY626146
*Ramaria flavescens*	TENN 36864	Germany	KY626140
*Ramaria flavescens*	ZT Myc, Schild 1517	Slovenia	MH322680
*Ramaria flavescentoides*	MH-2013	Pakistan	MG760617
*Ramaria flavicingula*	AMB 18607	France	MT452503
***Ramaria flavicoralloides***	**BJTC FM0970**	**China**	**PP537926**
***Ramaria flavicoralloides***	**BJTC FM2215**	**China**	**PP537927**
*Ramaria flavigelatinosa*	MA-Fungi 48083	Spain	AJ408357
*Ramaria flavinedulis*	GM19056	Patagonia	OP177717
*Ramaria flavinedulis*	GM19117	Patagonia	OP177718
*Ramaria flavissima*	ZT Myc 57157	Spain	MT055966
*Ramaria flavobrunnescens*	iNat63604655	USA	ON479744
*Ramaria flavoides*	MA-Fungi 47972	Spain	AJ408380
*Ramaria flavoides*	MA-Fungi 47971	Spain	AJ408381
*Ramaria flavosalmonicolor*	ZT Myc 54973	Switzerland	KY626135
*Ramaria flavosalmonicolor*	AH 47740	Spain	MH322681
*Ramaria flavosaponaria*	AHB3	USA	MT196969
*Ramaria formosa*	OSC 1064203	USA	EU525994
*Ramaria formosa*	MA-Fungi 48087	Spain	AJ408393
*Ramaria formosa*	AMB 18199	Italy	KY626155
***Ramaria formosoides***	**BJTC FM2403**	**China**	**PP537938**
***Ramaria formosoides***	**BJTC FM2410**	**China**	**PP537939**
***Ramaria formosoides***	**BJTC FM3437**	**China**	**PP537941**
***Ramaria formosoides***	**BJTC FM3448**	**China**	**PP537942**
***Ramaria formosoides***	**BJTC FM3569**	**China**	**PP537940**
***Ramaria formosoides***	**BJTC FM3573**	**China**	**PP537943**
*Ramaria fumigata*	AH:47767	Spain	MH322669
*Ramaria fumigata*	NIFoS2370	Korea	KX814451
*Ramaria fumosiavellanea*	WTU-F-063048	USA	MK169345
*Ramaria gelatiniaurantia*	OSC 65737	USA	KP658144
*Ramaria gelatiniaurantia*	WTU-F-063049	USA	KX574474
*Ramaria gracilioides*	AMB 18543	Italy	NR_174818
*Ramaria gracilis*	-	Mexico	EU258553
*Ramaria gracilis*	OSC 134659	USA	JX310399
*Ramaria ichnusensis*	AMB 18582	Italy	NR_174820
*Ramaria ignicolor*	MA-Fungi 47978	Spain	AJ408386
*Ramaria inedulis*	isolate 12648	Chile	OP177723
*Ramaria inedulis*	GM19047	Argentina	OP177722
*Ramaria inquinata*	ZT Myc 55614	Slovenia	KY626147
*Ramaria intimorosea*	ZT Myc 57155	Slovenia	KY626148
*Ramaria kafaensis*	FR-0246021	Äthiopien	MT542981
*Ramaria lacteobrunnescens*	MA-Fungi 48470	Spain	MH322682
*Ramaria largentii*	OSC 109294	USA	KP658126
*Ramaria largentii*	OSC 67012	USA	KP658130
*Ramaria leptoformosa*	SAT-21-290-06	USA	OP205430
*Ramaria leptoformosa*	WTU-F-063036	USA	MN809535
***Ramaria lingkongshanensis***	**BJTC FM1723**	**China**	**PP537934**
*Ramaria longispora*	iNat63913396	USA	ON479741
*Ramaria longispora*	iNAT:17234752	USA	OM522294
*Ramaria lorithamnus*	PDD 95771	New Zealand	HQ533039
*Ramaria luteoaurantiaca*	AMB 18525	Italy	NR_174815
*Ramaria luteovernalis*	MCVE 28637	Italy	KT357471
*Ramaria maculatipes*	OSC 69937	USA	KP658151
*Ramaria maculatipes*	WTU-F-063042	USA	KX574476
*Ramaria magnifica*	MA-Fungi 26386	Spain	AJ408354
*Ramaria magnipes*	WTU 063057	Spain	MK493040
*Ramaria magnipes*	WTU-F-063057	USA	MK169351
*Ramaria mediterranea*	MA-Fungi 39877	Spain	AJ408370
***Ramaria obtusa***	**BJTC FM3519**	**China**	**PP537944**
***Ramaria obtusa***	**BJTC FM3521**	**China**	**PP537945**
*Ramaria obtusissima*	TENN 69158	USA	KJ655554
*Ramaria ossolana*	AMB 1852	Italy	NR_174816
*Ramaria pallida*	IZS19097028/8-94	Italy	MZ005501
*Ramaria pallida*	AMB n. 18211	Italy	MF288931
*Ramaria pallidissima*	ZT Myc 55616	Italy	NR_171869
*Ramaria pallidosaponaria*	TENN 36849	Italy	KY626142
*Ramaria parabotrytis*	ZT Myc 58930	Spain	MH216040
*Ramaria parabotrytis*	AMB n. 18281	Spain	MH216039
*Ramaria paraconcolor*	AMB 18546	Italy	NR_174819
*Ramaria patagonica*	GM19095	Argentina	OP177714
*Ramaria patagonica*	GM19106	Argentina	OP177713
***Ramaria persicinoflava***	**BJTC FM1070**	**China**	**PP537923**
*Ramaria pinicola*	isolate 139.1	USA	DQ365649
***Ramaria platyrugosa***	**BJTC FM3526**	**China**	**PP537925**
*Ramaria polonica*	L 0053314	Spain	MT055947
*Ramaria praecox*	AH 47804	Spain	MF564298
*Ramaria primulina*	AMB 17480	Spain	MK493043
*Ramaria primulina*	TENN 038182	Spain	MK493042
*Ramaria pseudoflava*	AMB 17392	Spain	MK493046
*Ramaria pseudoflava*	AMB 17390	Spain	MK493045
*Ramaria pseudogracilis*	MA-Fungi 48067	Spain	AJ408384
*Ramaria pumila*	MA-Fungi 47983	Spain	AJ408388
*Ramaria rainierensis*	KA12-1702	South Korea	KR673634
*Ramaria rainierensis*	WTU-F-063041	USA	KX574466
*Ramaria rasilispora*	S12B1602	USA	KU574735
*Ramaria rasilispora*	S12B1601	USA	KU574736
*Ramaria rasilisporoides*	iNat62919302	USA	ON479762
*Ramaria rasilisporoides*	WTU-F-043029	USA	MK169346
*Ramaria rielii*	AMB n. 17268	Italy	MK100905
*Ramaria rielii*	AMB n. 17163	Italy	MK046801
*Ramaria rubella*	OSC 140659	USA	JX310405
*Ramaria rubella*	OSC 110600	USA	KC346860
*Ramaria rubiginosa*	Isolate 170.3	USA	DQ365650
*Ramaria rubribrunnescens*	CB 08258	Mexico	KT307881
*Ramaria rubribrunnescens*	CB 08392	Mexico	KT307882
*Ramaria rubribrunnescens*	OSC 119676	USA	EU652352
*Ramaria rubribrunnescens*	OSC 140575	USA	JX310406
*Ramaria rubribrunnescens*	OSC 66051	USA	KY354750
*Ramaria rubribrunnescens*	OSC 131274	USA	EU837231
*Ramaria rubribrunnescens*	OSC 105345	USA	KP658107
*Ramaria rubricarnata*	iNat82256995	USA	ON975053
*Ramaria rubricarnata*	WTU F 043061	Spain	MT055913
*Ramaria rubrievanescens*	AH 47481	Spain	MH322689
*Ramaria rubrievanescens*	OSC 134654	USA	JX310408
*Ramaria rubrievanescens*	OSC 140637	USA	JX310409
*Ramaria rubripermanens*	OSC 130793	USA	JX310412
*Ramaria rubripermanens*	OSC 134755	USA	KY510828
*Ramaria rufescens*	AMB 18554	Spain	MT055936
*Ramaria rufescens*	AMB 18608	France	MT452504
*Ramaria sandaracina*	OSC 1064136	USA	EU525992
*Ramaria sandaracina*	WTU-F-063058	USA	KX574484
*Ramaria sanguinea*	MA-Fungi 48080	Spain	AJ408373
*Ramaria sanguinea*	AMB 18200	Italy	KY626159
*Ramaria sardiniensis*	ZT Myc 57149	Italy	KY626138
*Ramaria spinulosa*	MA-Fungi 47990	Spain	AJ292293
*Ramaria stricta*	MA-Fungi 33215	Spain	AF442097
*Ramaria stricta*	MA-Fungi 48068	Spain	AJ408372
*Ramaria stuntzii*	OSC 73315	USA	KP658122
*Ramaria stuntzii*	WTU-F-063050	USA	KX574478
*Ramaria subalpina*	KD-14-006	India	KT824242
*Ramaria subaurantiaca*	FLAS:F-70702-MES-4176	USA	OP339697
*Ramaria subbotrytis*	OSC 1064182	USA	EU525993
***Ramaria subcolumnaris***	**BJTC FM2198**	**China**	**PP537930**
***Ramaria subcolumnaris***	**BJTC FM1977**	**China**	**PP537928**
***Ramaria subcolumnaris***	**BJTC FM2607**	**China**	**PP537929**
*Ramaria subtilis*	AH 48020	Spain	MF564300
*Ramaria subtilis*	MA-Fungi 48010	Spain	MF564301
*Ramaria suecica*	MA-Fungi 48078	Spain	AJ408360
*Ramaria suecica*	DG 1218	USA	KU574731
*Ramaria synaptopoda*	WTU-F-063037	USA	KX574485
*Ramaria testaceoflava*	4441 SL	USA	KU574732
*Ramaria thalliovirescens*	AMB 18523	Spain	MT055904
*Ramaria thalliovirescens*	AMB 18519	Spain	MT055898
*Ramaria thiersii*	OSC 47006	USA	KP658143
*Ramaria thiersii*	OSC 112045	USA	KY354761
*Ramaria thindii*	CAL 1786	India	NR_171845
*Ramaria tsugina*	iNat63859415	USA	ON479753
*Ramaria tsugina*	WTU-F-043052	USA	KX574467
*Ramaria velocimutans*	WTU-F-063045	USA	KX574487
*Ramaria verlotensis*	WTU-F-063047	USA	KX574480
*Ramaria vinosimaculans*	OSC 73528	USA	KP658155
*Ramaria vinosimaculans*	WTU-F-063043	USA	KX574488
*Ramaria violacea*	AMB 18564	Spain	MT055944
*Ramaria violacea*	AMB 18563	Spain	MT055943
*Ramaria xanthosperma*	IMG5012-18	USA	MW680289
*Ramaria xanthosperma*	ALV16744	Spain	MK408625

Newly generated sequences are in bold.

**Table 2.** Specimens used in four combined loci (ITS-nrLSU-*atp6*-mtSSU) phylogenetic analysis and their GenBank accession numbers.

Taxon	Voucher ID	Country	ITS	nrLSU	*atp6*	mtSSU
*Beenakia fricta*	K2083	USA	–	AY574693	AY574833	AY574766
*Clavariadelphus ligula*	OSC67068	USA	–	AY574650	AY574793	AY574723
*Clavariadelphus occidentalis*	OSC37018	USA	–	AY574648	AY574791	AY574721
*Clavariadelphus truncatus*	OSC67280	USA	–	AY574649	AY574792	AY574722
*Gallacea scleroderma*	OSC59621	USA	–	AY574645	AY574787	AY574719
*Gautieria monticola*	OSC65121	USA	–	AY574651	AY574794	AY574724
*Gautieria parksiana*	OSC58907	USA	–	AY574652	AY574795	AY574725
*Gloeocantharellus dingleyae*	PDD: 30179	USA	–	AY574668	–	AY574741
*Gloeocantharellus novae-zelandiae*	PDD: 44960	USA	–	AY574666	AY574809	AY574739
*Gloeocantharellus pallidus*	BPI54917	USA	–	AY574673	AY574815	AY574746
*Gloeocantharellus papuanus*	PERTH06707114	USA	–	AY574667	AY574810	AY574740
*Gomphus brunneus*	BR034190-46	USA	–	AY574680	AY574821	AY574753
*Gomphus clavatus*	OSC97616	USA	–	AY574664	AY574807	AY574737
*Gomphus clavatus*	UPS	USA	–	AY574665	AY574808	AY574738
*Hysterangium coriaceum*	OSC64939	USA	–	AY574686	AY574826	AY574759
*Hysterangium crassum*	OSC110447	USA	–	AY574687	AY574827	AY574760
*Hysterangium occidentalis*	OSC47048	USA	–	AY574685	AY574825	AY574758
*Kavinia alboviridis*	O102140	USA	–	AY574692	AY574832	AY574765
*Kavinia himantia*	O102156	USA	–	AY574691	AY574831	AY574764
*Lentaria pinicola*	SUC46	USA	–	AY574689	AY574829	AY574762
*Lentaria pinicola*	SUC89	USA	–	AY574688	AY574828	AY574761
*Lentaria pinicola*	SUC560	USA	–	AY574690	AY574830	AY574763
*Mutinus elegans*	OSC107657	USA	–	AY574643	AY574785	AY574717
*Phaeoclavulina africana*	TENN39621	USA	–	AY574653	AY574796	AY574726
*Phaeoclavulina cokeri*	TENN36030	USA	–	AY574701	AY574843	AY574774
*Phaeoclavulina curta*	OSC8711	USA	–	AY574713	AY574858	–
*Phaeoclavulina cyanocephala*	TENN37827	USA	–	AY574710	AY574854	AY574779
*Phaeoclavulina eumorpha*	TENN36218	USA	–	AY574712	AY574856	AY574781
*Phaeoclavulina eumorpha*	TENN37842	USA	–	–	AY574857	AY574782
*Phaeoclavulina gigantea*	FH109	USA	–	AY574703	AY574845	AY574776
*Phaeoclavulina grandis*	BR073158-06	USA	–	AY574678	AY574820	AY574751
*Phaeoclavulina guyanensis*	FH84	USA	–	AY574706	AY574848	–
*Phaeoclavulina insignis*	FH104	USA	–	AY574704	AY574846	–
*Phaeoclavulina longicaulis*	TENN33826	USA	–	AY574700	AY574842	AY574773
*Phaeoclavulina ochraceo-virens*	OSC23475	USA	–	AY574714	AY574859	–
*Phaeoclavulina pancaribbea*	TENN31836	USA	–	AY574707	AY574849	–
*Phaeoclavulina subclaviformis*	BR073159-07	USA	–	AY574679	–	AY574752
*Phaeoclavulina viridis*	OSC97708	USA	–	AY574675	AY574817	AY574748
*Phaeoclavulina viridis*	FH1853	USA	–	AY574676	AY574818	AY574749
*Phaeoclavulina viridis*	PERTH4302	USA	–	AY574677	AY574819	AY574750
*Phallus impudicus*	OSC107655	USA	–	AY574642	AY574784	AY574716
*Protubera nothofagi*	OSC59699	USA	–	AY574644	AY574786	AY574718
***Ramaria affinis***	**BJTC HSA370**	**China**	**–**	**PP544920**	**–**	**PP550681**
***Ramaria apicaliochracea***	**BJTC FM2738**	**China**	**PP537936**	**PP544912**	**PP556462**	**PP550680**
***Ramaria apicaliochracea***	**BJTC FM1078**	**China**	**PP537935**	**PP544911**	**PP556461**	**PP550679**
*Ramaria apiculata*	OSC23549	USA	–	AY574695	AY574836	AY574768
*Ramaria apiculata* var. *brunne*	TENN53935	USA	–	AY574696	AY574837	AY574769
*Ramaria araiospora* var. *araiospora*	SUCM739	USA	–	AF213068	AY574838	AF213141
*Ramaria araiospora* var. *araiospora*	SUCM556	USA	–	AY574697	AY574839	AY574770
*Ramaria aurea*	AMB 18352	Spain	MN637783	MN637796	–	–
*Ramaria aurea*	AMB 18351	Spain	MN637782	MN637795	–	–
*Ramaria barenthalensis*	AMB 17381	Italy	MK493038	MK493049	–	–
***Ramaria barenthalensis***	**BJTC FM2750**	**China**	**PP537924**	**PP544897**	**–**	**PP550673**
*Ramaria botrytis* var. *botrytis*	SUCM457	USA	–	AY574698	AY574840	AY574771
*Ramaria botrytis* var. *botrytis*	SUCM740	USA	–	AY574699	AY574841	AY574772
***Ramaria cadmioaurantiaca***	**BJTC HSA288**	**China**	**PP537922**	**PP544895**	**PP556452**	**–**
***Ramaria cadmioaurantiaca***	**BJTC HSA280**	**China**	**PP537921**	**PP544894**	**PP556451**	**–**
***Ramaria cadmioaurantiaca***	**BJTC FM2242**	**China**	**PP537920**	**PP544893**	**PP556450**	**–**
*Ramaria circinans*	NYS1	USA	–	AY574702	AY574844	AY574775
*Ramaria circinans* var. *anceps*	SUCM615	USA	–	AY574711	AY574855	AY574780
***Ramaria conferta***	**BJTC HSA258**	**China**	**PP537937**	**PP544913**	**–**	**–**
***Ramaria cyanophila***	**BJTC FM2557**	**China**	**PP537933**	**PP544908**	**–**	**–**
***Ramaria cyanophila***	**BJTC HSA260**	**China**	**PP537931**	**PP544906**	**–**	**–**
***Ramaria cyanophila***	**BJTC HSA261**	**China**	**PP537932**	**PP544907**	**–**	**–**
*Ramaria flavescens*	TENN36864	Germany	KY626140	NG_241888	–	–
*Ramaria flavicingula*	AMB 18607	Spain	MT452503	MT434370	–	–
***Ramaria flavicoralloides***	**BJTC FM3520**	**China**	**–**	**PP544902**	**PP556457**	**PP550678**
***Ramaria flavicoralloides***	**BJTC FM0970**	**China**	**PP537926**	**PP544899**	**PP556454**	**PP550675**
***Ramaria flavicoralloides***	**BJTC FM2215**	**China**	**PP537927**	**PP544900**	**PP556455**	**PP550676**
***Ramaria flavicoralloides***	**BJTC FM2790**	**China**	**–**	**PP544901**	**PP556456**	**PP550677**
*Ramaria formosa*	AMB n 18199	Italy	KY626155	KY626156	–	–
***Ramaria formosoides***	**BJTC FM2403**	**China**	**PP537938**	**PP544914**	**–**	**–**
***Ramaria formosoides***	**BJTC FM2410**	**China**	**PP537939**	**PP544915**	**–**	**–**
***Ramaria formosoides***	**BJTC FM3437**	**China**	**PP537941**	**PP544917**	**–**	**–**
***Ramaria formosoides***	**BJTC FM3448**	**China**	**PP537942**	**PP544918**	**–**	**–**
***Ramaria formosoides***	**BJTC FM3569**	**China**	**PP537940**	**PP544916**	**–**	**–**
***Ramaria formosoides***	**BJTC FM3573**	**China**	**PP537943**	**PP544919**	**–**	**–**
*Ramaria gelatiniaurantia* var. *violeitingens*	SUCM830	USA	–	AY574708	AY574851	AY574777
*Ramaria largentii*	OSC109294	USA	KP658126	KP637054	–	–
*Ramaria largentii*	OSC67012	USA	KP658130	KP637058	–	–
***Ramaria lingkongshanensis***	**BJTC FM1723**	**China**	**PP537934**	**PP544909**	**–**	**–**
***Ramaria lingkongshanensis***	**BJTC FM1724**	**China**	**–**	**PP544910**	**–**	**–**
*Ramaria luteoaurantiaca*	AMB 18525	Spain	NR_174815	NG_079662	–	–
*Ramaria moelleriana*	OSC36422	USA	–	DQ218619	DQ218908	–
***Ramaria obtusa***	**BJTC FM3521**	**China**	**PP537945**	**PP544922**	**–**	**–**
***Ramaria obtusa***	**BJTC FM3519**	**China**	**PP537944**	**PP544921**	**–**	**–**
*Ramaria ossolana*	AMB18527	Italy	NR_174816	NG_079663	–	–
*Ramaria pallidissima*	ZT Myc 55616	Italy	NR_171869	NG_075339	–	–
*Ramaria pallidosaponaria*	TENN36849	Italy	KY626142	KY626143	–	–
*Ramaria persicina*	RP32	Mexico	–	MZ435312	–	MZ435924
***Ramaria persicinoflava***	**BJTC FM1070**	**China**	**PP537923**	**PP544896**	**PP556453**	**–**
***Ramaria platyrugosa***	**BJTC FM3526**	**China**	**PP537925**	**PP544898**	**–**	**PP550674**
*Ramaria radicans*	RP92	Mexico	–	MZ435314	–	MZ435926
*Ramaria rainieriensis*	SUCM431	USA	–	AY574694	AY574835	AY574767
*Ramaria rainieriensis*	SUCM231	USA	–	AF213115	AY574834	AF213135
*Ramaria rubribrunnescens*	SUCM844	USA	–	AF213098	AY574852	AF213142
*Ramaria rubricarnata*	WTU F 43061	USA	MT055913	MT053204	–	–
*Ramaria stuntzii*	SUC214	USA	–	AF213102	AY574850	AF213134
*Ramaria suaveolens*	RS32	Mexico	–	MZ435315	–	MZ435927
***Ramaria subcolumnaris***	**BJTC FM2198**	**China**	**PP537930**	**PP544905**	**PP556460**	**–**
***Ramaria subcolumnaris***	**BJTC FM1977**	**China**	**PP537928**	**PP544903**	**PP556458**	**–**
***Ramaria subcolumnaris***	**BJTC FM2607**	**China**	**PP537929**	**PP544904**	**PP556459**	**–**
*Ramaria suecica*	BPI1	USA	–	AY574705	AY574847	–
*Ramaria thiersii*	OSC47006	USA	KP658143	KP637072	–	–
*Ramaria vinosimaculans*	OSC23287	USA	–	AY574709	AY574853	AY574778
*Turbinellus flabellatus*	K1770	USA	–	AY574681	AY574822	AY574754
*Turbinellus floccosus*	UC924302	USA	–	AY574663	AY574806	AY574736
*Turbinellus floccosus*	OSA-MY-1840	USA	–	AY574655	AY574798	AY574728
*Turbinellus floccosus*	OSC69167	USA	–	AY574656	AY574799	AY574729
*Turbinellus floccosus*	TENN33233	USA	–	AY574657	AY574800	AY574730
*Turbinellus floccosus*	TENN33295	USA	–	AY574659	AY574802	AY574732
*Turbinellus floccosus*	MICH5588	USA	–	AY574660	AY574803	AY574733
*Turbinellus floccosus*	MICH10721	USA	–	AY574661	AY574804	AY574734
*Turbinellus floccosus*	UC759902	USA	–	AY574662	AY574805	AY574735
*Turbinellus fujisanensis*	OSA-MY-1842	USA	–	AY574669	AY574811	AY574742
*Turbinellus kauffmanii*	OSC97590	USA	–	AY574672	AY574814	AY574745
*Turbinellus floccosus*	OSA-MY-1839	USA	–	AY574654	AY574797	AY574727
*Turbinellus floccosus*	SFSU21238	USA	–	AY574658	AY574801	AY574731
*Turbinellus fujisanensis*	OSA-MY-1841	USA	–	AY574670	AY574812	AY574743
*Turbinellus kauffmanii*	MICH10069	USA	–	AY574671	AY574813	AY574744

Newly generated sequences are in bold.

Maximum Likelihood (ML) was performed using RAxML v8.0.14 (Stamatakis 2014) for all two datasets by running 1,000 bootstrap replicates under the GTRGAMMAL model. Bayesian Inference (BI) was conducted using MrBayes v3.1.2 (Ronquist and Huelsenbeck 2003). In the BI analysis, after selecting the best substitution model (GTR + I + G for ITS in single gene dataset, nrLSU and *atp6* in combined dataset; GTR + G for ITS and mtSSU in combined dataset) determined by MrModeltest v2.3 (Nylander 2004), two independent runs of four chains were conducted for 10,000,000 Markov Chain Monte Carlo (MCMC) generation using the default settings and sampled every 100 generations. The temperature value was lowered to 0.20, burn-in was set to 0.25, and the run was automatically stopped as soon as the average standard deviation of split frequencies reached below 0.01. A 50% majority-rule consensus tree was constructed.

Clades with bootstrap support (BS) ≥ 70% and Bayesian posterior probability (PP) ≥ 0.99 were considered significantly supported (Hillis and Bull 1993; Alfaro 2003). All phylogenetic trees were viewed with TreeView v1.0.0.0 (Page 2001).

## 3. Results

### 3.1. Phylogenetic analysis

In the two datasets used in our phylogenetic analyses, 78 sequences (26 ITS, 30 nrLSU, 13 *atp6*, and 9 mtSSU) newly generated from 30 collections were included. The final combined dataset (ITS-nrLSU-*atp6*-mtSSU) included 239 sequences (40 ITS, 118 nrLSU, 83 *atp6*, and 77 mt-SSU) and was comprised of 342 characters for ITS, 529 characters for nrLSU, 556 characters for *atp6*, 399 characters for mtSSU after exclusion of poorly aligned sites. The ITS dataset included 225 sequences resulting in an alignment with 412 characters after exclusion of poorly aligned sites. ML and BI analyses yielded similar tree topologies and only the trees inferred from the ML analysis are therefore shown (Figure 1 for the combined dataset, Figure 2 for ITS dataset).

**Figure 1.**
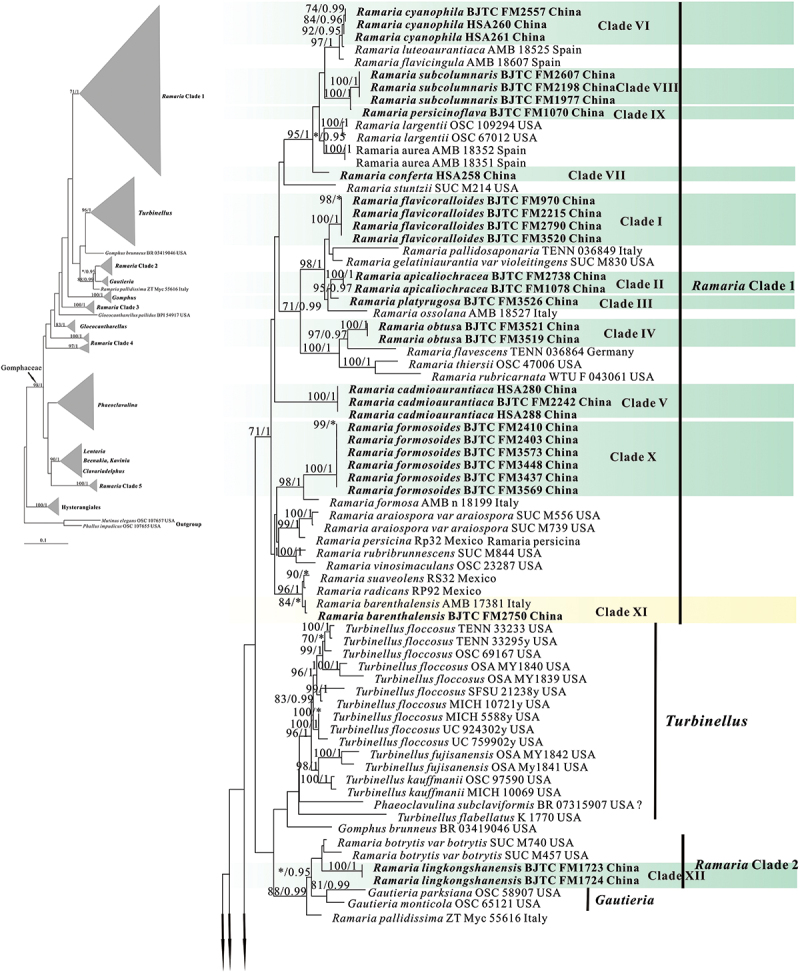
Phylogenetic tree generated from a maximum likelihood analysis based on combined its-nrLSU-*atp6*-mtSSU sequences, showing the phylogenetic relationships of *Ramaria*. Numbers representing likelihood bootstrap support (MLBS ≥ 70%, left) and significant Bayesian posterior probability (BPP ≥ 0.99, right) are indicated above the nodes. Sequences from Shanxi Province, northern China are printed in bold.

**Figure 1.**
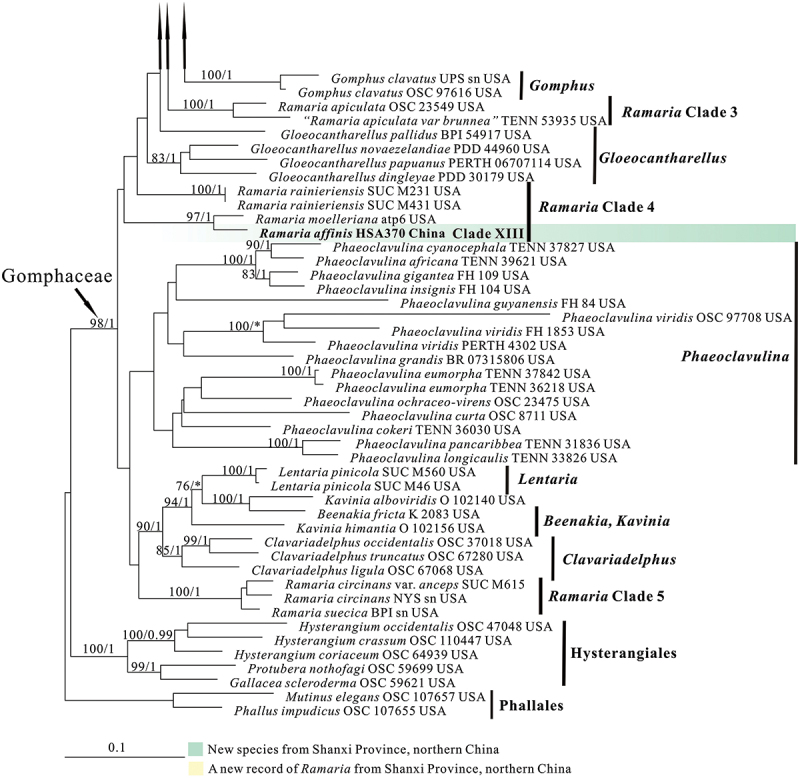
(Continued).

**Figure 2.**
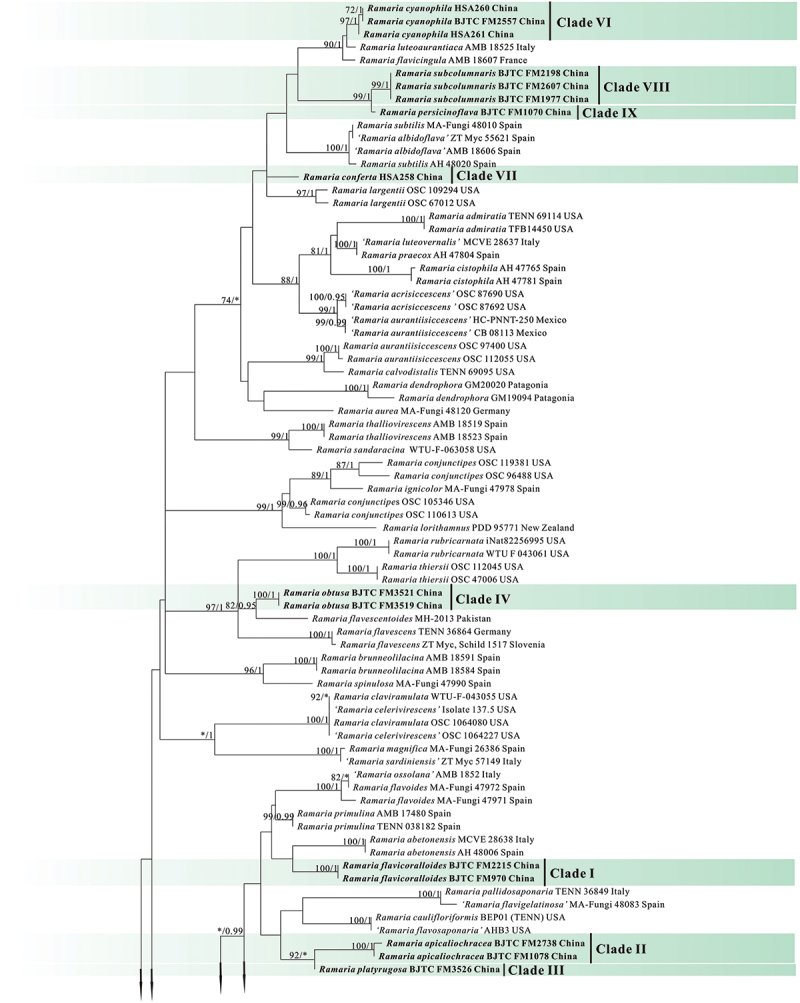
Phylogenetic tree generated from a maximum likelihood analysis based on ITS sequences, showing the phylogeneticrelationships of *Ramaria*. Numbers representing likelihood bootstrap support (MLBS ≥ 70%, left) and significant Bayesian posteriorprobability (BPP ≥ 0.99, right) are indicated above the nodes. Sequences from Shanxi Province, northern China are printed in bold.

**Figure 2.**
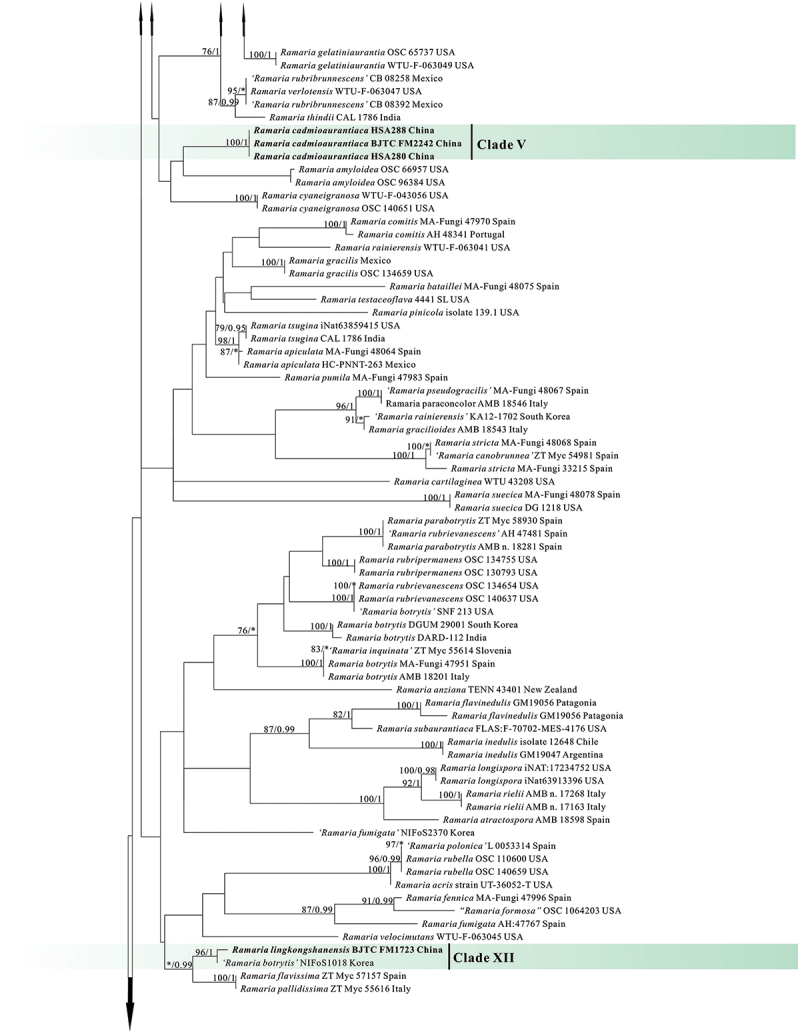
(Continued).

**Figure 2.**
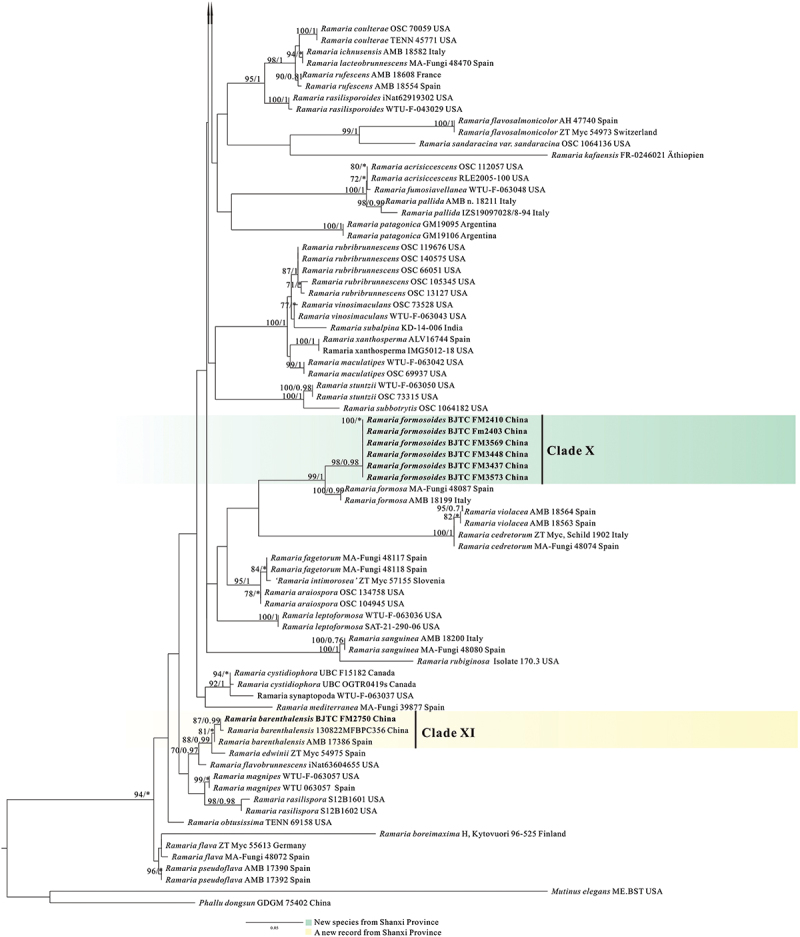
(Continued).

**Figure 3.**
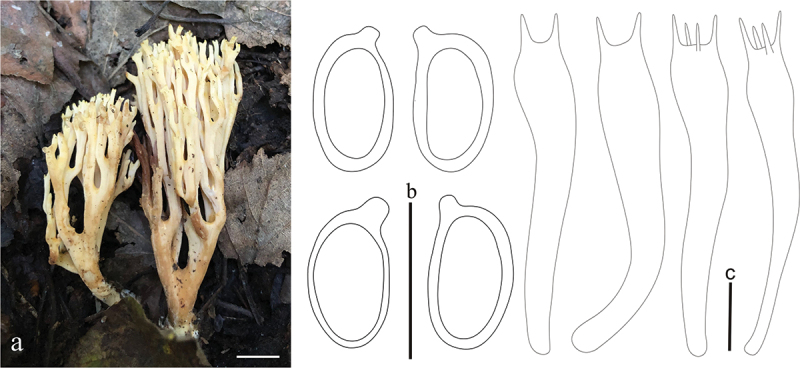
*Ramaria affinis*. (a) Basidiomata. (b) Basidiospores. (c) Basidia. Scale bars: a = 2 cm, b – c = 10 μm.

Our phylogenetic analysis based on the combined dataset revealed that the *Ramaria* species formed five major clades, and scattered in the Gomphaceae clade, indicating *Ramaria* was a paraphyletic genus (Figure 1), which is consistent with that obtained in previous studies, such as Humpert et al. (2001), Giachini et al. (2010), and González et al. (2023). Our 30 collections were resolved in 13 strong support clades (Marked as Clade I−XIII). Of them, 12 clades were independent, suggesting they represented 12 species and were new found species. We therefore described these probably novel species in this paper (See Taxonomy). The remaining one (Clade XI) contained the authentic sequences of *Ramaria barenthalensis* Franchi & M. Marchetti, which implied our collection (BJTC FM2750) should be identified as *R. barenthalensis*, and it is a new report to China. In ITS-based tree (Figure 2), 12 clades mentioned above were recognised, and Clade XI represented *R. barenthalensis*. Clade XIII was not included as the ITS sequence of the voucher specimen (HSA 370) was not successfully sequenced. Moreover, Clade II and Clade III were sisters to each other (Figures 1 and 2); Clade IV was sister to *Ramaria flavescens* (Schaeff.) R.H. Petersen (Figure 1) or *Ramaria flavescentoides* Hanif & Khalid (Figure 2); Clade VI was sister to *Ramaria luteoaurantiaca* Franchi & M. Marchetti and *Ramaria flavicingula* R.H. Petersen (Figures 1 and 2); Clade VIII and Clade IX were sisters (Figures 1 and 2), and then clustered a moderate support clade together with the clade compose of *Ramaria albidoflava* Schild and *Ramaria subtilis* (Coker) Schild in ITS-based tree (Figure 2); Clade X was sister to *Ramaria formosa* (Pers.) Quél. (Figures 1 and 2).

### 3.2. Taxonomy

***Ramaria*** ***affinis*** L. Fan, Y. Li & N. Mao, **sp. nov**. Figure 3

MycoBank: MB853042.

Etymology: *affinis*, referring to the close relationship to *Ramaria moelleriana* (Bres. & roum.) corner.

Typification: China, Shanxi Province, Lvliang city, Xing county, Heicha Mountain, on the ground in broadleaf forest dominated by *Betula* spp., 1,680 m elev., 5 September 2018, J.Z. Cao, LH370 (holotype HSA 370). GenBank: nrLSU = PP544920; mtSSU = PP550681.

Basidiomata solitary, 10 − 13 cm high, 4.5 − 5 cm broad, repeatedly branched dichotomously in 3 – 4 ranks, coralloid, usually pale yellow (#f3efaa); Stipe compound to fasciculate in groups of 2 – 3, pale ochraceous (#ecc07d) when fresh, brown (#d9a36c) after drying, tapering gradually downward into a tangle of white mycelia; Context white (#ffffff), solid, brittle when dry, negative when exposed to ferric sulphate; Major branches 3 − 4, ascending, terete, pale yellow; Branches 3 − 4 ranks, ascending, internodes diminishing gradually upward, pale yellow (#f3efaa) below; Apices acute, pale brown (#ecc07d) to brownish-green (#a1966b); Taste and odour reactions not recorded.

Hyphal system monomitic, generative hyphae simple-septate, branched, walls smooth and hyaline; Stipe tramal hyphae 3.7 − 7.5 µm wide, thin-walled, parallel, hyaline, clamped; Gloeoplerous hyphae not observed; Tramal hyphae of upper branches 2.5 − 7.5 µm wide, and inflated up to 16 µm, thin-walled, parallel, hyaline, clamped; Rhizomorph hyphae of basal mat 1.2 − 7.5 µm diam., thin-walled, parallel, hyaline, clamped, crystals absent; Hymenium all along the basidiomata; Basidia 45 − 60 × 7.5 − 10 µm, clavate, simple-septate, clampless, multiguttulate when mature, strongly cyanophilous, 2/4-spored, sterigmata 3.5 − 5 µm long; Basidiospores [30/2/1] 7.5 − 8.75 (−9.7) × 3.75 − 5 µm, [Q=(1.45−)1.5 − 2.1(−2.3), Q_m_ = 1.8 ± 0.24], elliptical, pale yellow under bright field, smooth, inamyloid, with 1-several guttulate, strongly cyanophilous.

Habitat: Solitary on the ground in broadleaf forest dominated by *Betula* spp.

Distribution: Currently only known from Shanxi Province, northern China.

Notes: *Ramaria affinis* is characterized by pale yellow basidiomata, clamped hyphae, and clampless basidia. *Ramaria affinis* is phylogenetically closely related to *R. moelleriana* (Figure 1), however, *R. moelleriana* is distinguished by its pale flesh apices, flat and reddish brown branches, and easily collapsed basidia (Corner 1950).

**Figure 4.**
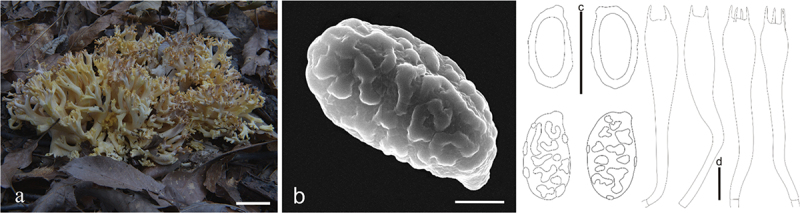
*Ramaria apicaliochracea*. (a) Basidiomata. (b) Scanning electron microscope (SEM) photographs of basidiospores. (c) Basidiospores. (d) Basidia. Scale bars: a = 2 cm, b = 2 μm, c – d = 10 μm.

***Ramaria*** ***apicaliochracea*** L. Fan, Y. Li & N. Mao, **sp. nov**. Figure 4

MycoBank: MB853043.

Etymology: *apicaliochracea*, referring to the color of branch-apex of basidiomata.

Typification: China, Shanxi Province, Qinshui County, Shangwoquan Village, on the ground in broadleaf forest dominated by *Quercus* spp., 1,170 m elev., 8 September 2022, J.Z. Cao, LH1697 (BJTC FM2738). GenBank: ITS = PP537936; nrLSU = PP544912; mtSSU = PP550680; atp6 = PP556462.

Basidiomata solitary, up to 7 cm high, 15 cm broad, repeatedly branched dichotomously in 4 – 5 ranks, coralloid, usually obconic in profile, pale yellow (#fff095) to pale brown (#cb945c); Stipe 1 − 2 cm high, fasciculate, cream (#fff9d0) to pale yellow (#fff4a6), tapering gradually downward into a tangle of white mycelia, with a few abortive stumps high on stipe; Context white (#ffffff), solid, brittle when dry, negative when exposed to ferric sulphate; Major branches 4 − 5, stout, ascending, more and less terete, cream (#fff9d0) to pale yellow (#fff4a6); Branches 4 − 5 ranks, ascending, smooth surface, cream (#fff9d0) to pale yellow (#fff4a6), polychotomous, internodes diminishing gradually upwards; Axils more or less rounded; Apices acute, rather crowded, pale yellow (#fff18d) when young, ochreous (#e7ab6e) by maturity; Taste and odour not recorded.

Hyphal system monomitic, generative hyphae simple-septate, branched, walls smooth and hyaline; Stipe tramal hyphae 3 − 6 µm wide, thin-walled, interwoven, hyaline, clampless; Gloeoplerous hyphae not observed; Tramal hyphae of upper branches 2 − 6 µm wide, thin-walled, loosely parallel, hyaline, clampless; Rhizomorph hyphae of basal mat 2 − 5 µm diam., thin-walled, clampless, crystals absent; Hymenium all along the basidiomata; Basidia 50 − 65(−70) × 9 − 10 µm, clavate, simple-septate, clampless, multiguttulate, strongly cyanophilous, 2/4-spored, sterigmata 3 − 5 µm long; Basidiospores [60/2/2] (8−)9 − 11(−12) × 4 − 5 µm, [Q = (1.6−)1.8 − 2.75(−3), Q_m_ = 2.17 ± 0.35], ellipsoid, very light yellow under bright field, slightly thick-walled, ornamented with scattered small warts, warts very low, irregular-shaped to short ridged, inamyloid, with 1-several guttulae, strongly cyanophilous.

Habitat: Solitary on the ground in broadleaf forest dominated by *Quercus* spp.

Distribution: Currently only known from Shanxi Province, northern China.

Additional specimen examined: China, Shanxi Province, Qinshui County, Shangwoquan Village, on the ground in broadleaf forest dominated by *Quercus* spp., 1,170 m elev., 25 August 2020, J.Z. Cao, LH1096 (BJTC FM1078).

Notes: *Ramaria apicaliochracea* is recognized by its pale yellow to white color, branches with smooth surface, and clampless hyphae. The new species formed a strongly supported sister lineage to *R. platyrugosa*, and both resemblespores and clampless hyphae. However, the branches with smooth surface, and pale to cream stipe distinguish it from *R. platyrugosa*, which has flat and rugulose branches and pale brown stipe. The nrLSU sequences of *R. platyrugosa* share less than 98.7% similarity with those of *R.* *apicaliochracea*.

***Ramaria barenthalensis*** Franchi & M. Marchetti, Riv. Micol. 61: 199 (2019) Figure 5

**Figure 5.**
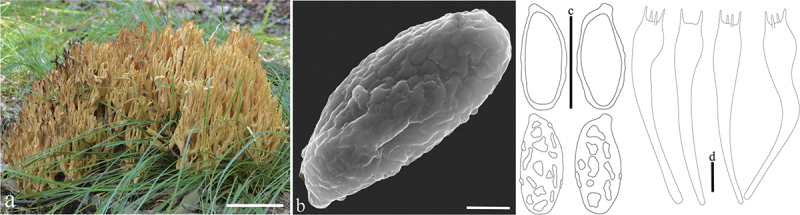
*Ramaria barenthalensis*. (a) Basidiomata. (b) Scanning electron microscope (SEM) photographs of basidiospores. (c) Basidiospores. (d) Basidia. Scale bars: a = 2 cm, b = 2 μm, c – d = 10 μm.

Basidiomata solitary, 5 − 12 cm high, 4 − 9 cm broad, brittle, repeatedly branched dichotomously in 3 – 4 ranks, coralloid, light brown (#ffcb81) when young and dark brown (#d07800) at maturity; Stipe variable in length and width, sometimes reduced to well-developed, fasciculate, smooth upward, light brown (#ffcb81) to cream (#fffeea), brown (#d07800) on drying, tapering gradually downward into a tangle of white mycelia; Context white (#ffffff) to light brown (#f9e8d8), soft, brittle when dry, negative when exposed to ferric sulphate; Branches 3 − 4 ranks, vertically oriented, furcated, elongated to flattened, smooth, light brown (#ffcb81) when young and dark brown (#d07800) at maturity; Apice finger-like, light brown (#ffcb81), brown (#d07800) on drying; Taste and odour not recorded.

Hyphal system monomitic, generative hyphae simple-septate, branched, walls smooth and hyaline; Stipe tramal hyphae 2.5 − 12.5 µm wide, thin-walled, parallel, hyaline, clamped; Gloeoplerous hyphae not observed; Tramal hyphae of upper branches 5 − 10 µm wide, thin-walled, loosely parallel, hyaline, clamped; Rhizomorph hyphae of basal mat 2.5 − 10 µm wide, thin-walled, loosely parallel, clamped, crystals absent; Hymenium all along the basidiomata; Basidia 50 − 67.5 × 9.5 − 10 µm, clavate, simple-septate, clampless, multiguttulate, cyanophilous, 2/4-spored, sterigmata 2.5 − 5 µm long. Basidiospores [30/1/1] (10−)11.2 − 13(−14.5) × (3.8−)4.5 − 5 µm [Q = (2.15−)2.25 − 2.55(−2.7), Qm = 2.47 ± 0.24], elliptical to ovoid, lightly yellow under bright field, ornamented with scattered irregular fine warts, inamyloid, with 1-several guttulae, cyanophilous.

Habitat: Solitary on the ground in the Coniferous and broad-leaved mixed forest dominated by *Quercus* spp. and *Pinus* spp.

Distribution: Known from northern China, Italy, and Pakistan.

Specimens examined: China, Shanxi Province, Fenyang County, baihuling forest farm, on the ground in broadleaf forest dominated by *Quercus* spp., 1,400 m elev., 1 September 2022, J.Z. Cao, CF1155 (BJTC FM2750).

Notes: This is the first report about *R. barenthalensis* in China. *Ramaria barenthalensis* is characterized by its light brown basidiomata, fasciculate stipe, white and pale brown context, and clamped hyphae. *Ramaria barenthalensis* phylogenetically closely related to *R. edwinii* Franchi & M. Marchetti, *R. flavobrunnescens* (G.F. Atk.) corner, *R. radicans* Cázares & G. Guevara, and *R. suaveolens* Cázares (Figures 1 and 2). *Ramaria edwinii* is distinguished from *R. barenthalensis* by its pale pinkish purple or pale pink context, which is positive when exposed to ferric sulphate and clamped basidia (Schild 1991; Franchi and Marchetti 2022); *Ramaria flavobrunnescens* by its context that moderately positive when exposed to ferric sulphate, clamped basidia and ochraceous basidiospores (Corner 1950); *Ramaria radicans* by its larger basidiomata (12 − 17 × 10 − 15 cm), cream to pale yellow upper branches, and clamped basidia; *Ramaria suaveolens* by its pale yellow to cream upper branches and branch apices that bruising brownish and clamped basidia (Cázares et al. 2011).

**Figure 6.**
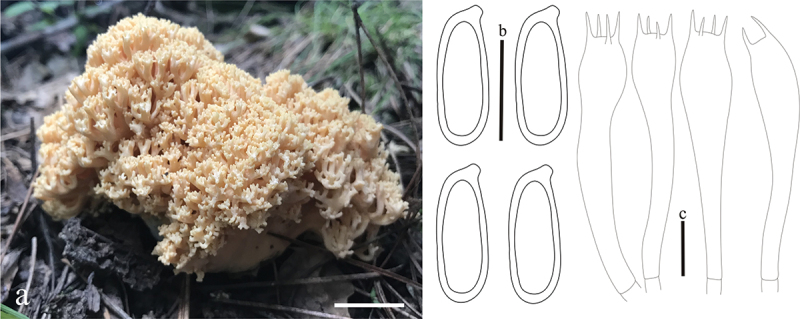
*Ramaria cadmioaurantiaca*. (a) Basidiomata. (b) Basidiospores. (c) Basidia. Scale bars: a = 2 cm, b – c = 10 μm.

***Ramaria*** ***cadmioaurantiaca*** L. Fan, Y. Li & N. Mao, **sp. nov**. Figure 6

MycoBank: MB853044.

Etymology: *cadmioaurantiaca*, referring to the color of basidiocarp.

Typification: China, Shanxi Province, Jinzhong City, Jiaocheng County, Haojiagou, 111°28’34’’E, 37°49’5’’N, elev. 1,800 m, 29 August 2018, collected by H. Liu LH288 (HSA 288). GenBank: ITS = PP537922; nrLSU = PP544895; atp6 = PP556452.

Basidiomata solitary, 5.5 − 11 cm high, 5 − 11.5 cm broad, brittle, repeatedly branched dichotomously in 4 – 5 ranks, coralloid, usually obconic to obovate in outline, saffron-yellow (#efd26a); Stipe short, 1 − 1.2 cm high, fasciculate, smooth upward, white (#fafafa) below, upward saffron-yellow (#efd26a), tapering gradually downward into a tangle of white mycelia, unchanging on drying, without abortive stumps on stipe; Context white (#ffffff), solid, brittle when dry, negative when exposed to ferric sulphate. Major branches 2 − 3, stout, ascending, more or less terete, saffron-yellow (#efd26a); Branches 4 − 5 ranks, ascending, more or less terete, with smooth walls, polychotomous, internodes diminishing gradually upward; Axils more or less rounded; Apices 1 − 2 mm long, obtuse, saffron−yellow (#efd26a); Taste and odour not recorded.

Hyphal system monomitic, generative hyphae simple-septate, branched, walls smooth and hyaline; Stipe tramal hyphae 2 − 12 µm wide, branched, thin-walled, parallel, hyaline, clampless; Gloeoplerous hyphae not observed; Tramal hyphae of upper branches 2.5 − 10 µm wide, thin-walled, loosely parallel, hyaline, clamped; Rhizomorph hyphae of basal mat 2 − 6 µm diam., thin-walled, loosely parallel, hyaline, clamped, crystals absent; Hymenium all along the basidiomata; Basidia (40−) 46 − 53 (−57) × 7 − 9 µm, clavate, simple-septate, clampless, multiguttulate, strongly cyanophilous, 4-spored, sterigmata 5 − 7.5 µm long. Basidiospores [90/3/3] (8−)9 − 12 × (3.5−) 4 − 5 µm [Q = (1.6−)2 − 2.75, Q_m_ = 2.36 ± 0.29], ellipsoid or elongate, pale yellow under bright field, slightly thick-walled, smooth, inamyloid, apiculate, with 1-several guttulae, moderately cyanophilous.

Habitat: Solitary or scattered on the ground in the coniferous and broad-leaved mixed forest dominated by *Quercus* spp. and *Pinus* spp.

Distribution: Currently known from Shanxi Province of northern China and Yunnan Province of southwestern China.

Additional specimens examined: China, Shanxi Province, Jinzhong City, Jiaocheng County, Haojiagou, on the ground in coniferous and broad-leaved mixed forest dominated by *Quercus* spp. and *Pinus* spp., 111°28’34’’E, 37°49’5’’N, elev. 1,800 m, 29 August 2018, collected by H. Liu LH280 (HSA 280); Yuanqu County, Lishan Mountain, Zhongcun Forest Farm, on the ground in broad leaved forest dominated by *Quercus* spp., 112°02’97’’E, 35°48’37’’N, elev. 1,672 m, 10 August 2022, collected by N. Mao MNM572 (BJTC FM2242).

Notes: *Ramaria cadmioaurantiaca* is recognized by its saffron-yellow basidiomata and rarely clamped hyphae. It is phylogenetically closely related to *R. rubribrunnescens* marr & D.E. Stuntz, and morphologically both of them share similar basidiomata color. However, *R. rubribrunnescens* has more branching pattern of basidiomata (up to 9 ranks from the stipe) and clampless hyphae (Marr and Stuntz 1973), which distinguish it from *R.* *cadmioaurantiaca*.

**Figure 7.**
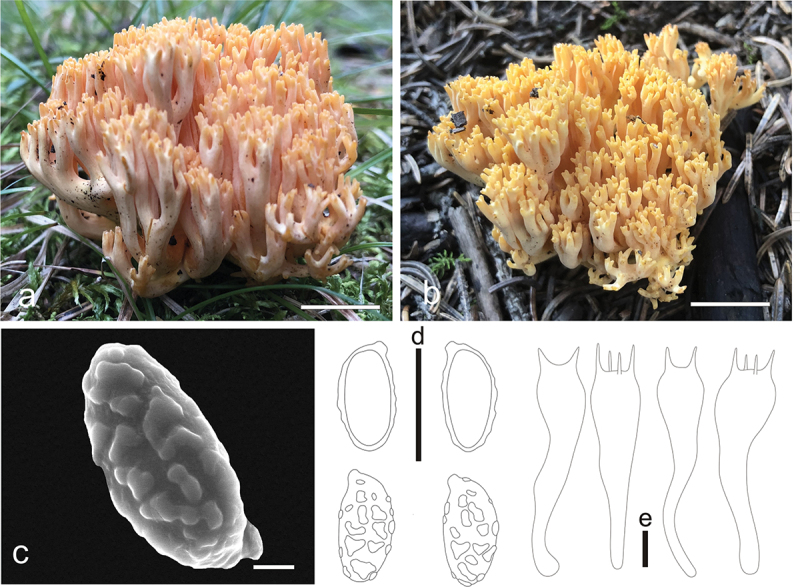
*Ramaria conferta*. (a – b) Basidiomata. (c) Scanning electron microscope (SEM) photographs of basidiospores. (d) Basidiospores. (e) Basidia. Scale bars: a – b = 2 cm, c = 2 μm, d – e = 10 μm.

***Ramaria*** ***conferta*** L. Fan, Y. Li & N. Mao, **sp. nov**. Figure 7

MycoBank: MB853045.

Etymology: *conferta*, referring to the crowded branch-apex of basidiomata.

Typification: China, Shanxi Province, Lvliang City, Jiaocheng County, Pangquangou, Badaogou, on the ground in coniferous and broadleaved mixed forest dominated by *Betula* spp. and *Pinus* spp., 2,000 m elev., 28 August 2018, J.Z. Cao, LH258 (HSA 258). GenBank: ITS = PP537937; nrLSU = PP544913.

Basiadioma solitary, 6.5 cm high, 8.5 cm broad, repeatedly branched dichotomously in 4 – 5 ranks, coralloid, usually light yellow (#ffd862) to light orange (#ffc14e), pale ochraceous buff (#dd9953) on drying; Stipe fasciculate, smooth upward, pale yellow (#ffd862) when fresh, pale brown (#dd9953) after drying, tapering gradually downward into a tangle of white mycelia; Context white (#ffffff), solid, brittle when dry, negative when exposed to ferric sulphate; Branches 4 − 5 ranks, stout, ascending, more or less terete, pale yellow (#ffd862) below, upward pale orange (#ffc14e); Axils more or less rounded; Apices obtuse, rather crowded, pale orange (#ffc14e); Taste and odour reactions not recorded.

Hyphal system monomitic, generative hyphae simple-septate, branched, walls smooth and hyaline; Stipe tramal hyphae 2 − 5 µm wide, thin-walled, loosely parallel, hyaline, clampless; Gloeoplerous hyphae not observed; Tramal hyphae of upper branches 2 − 9 µm wide, thin-walled, loosely parallel, hyaline, clampless; Rhizomorph hyphae of basal mat thin-walled, loosely parallel, hyaline, without crystals; Hymenium all along the basidiomata; Basidia 50 − 58 × 11 − 14 µm, clavate, simple-septate, clampless, strongly cyanophilous, 2/4-spored, sterigmata 4 − 6 µm long; Basidiospores [30/1/1] (8−)9 − 11 × 4 − 5 µm, [Q = 2 − 2.75, Q_m_ = 2.35 ± 0.28], ellipsoid, pale yellow under bright field, ornamented with scattered small warts, warts low, irregularly shaped, inamyloid, with 1-several guttulae, strongly cyanophilous.

Habitat: Solitary on the ground in coniferous and broadleaved mixed forest dominated by *Betula* spp. and *Pinus* spp.

Distribution: Currently only known from Shanxi Province, northern China.

Notes: *Ramaria conferta* is characterized by the light yellow to light orange basiadiomata, clampless hyphae, and basidia. *Ramaria conferta* is morphologically and phylogenetically closely related to *Ramaria largentii* marr & D.E. Stuntz and shares similar basidiomatal color and outline. However, *R. largentii* has larger basidiospores (11 − 15 × 3.5 − 5 µm) and clamped hyphae and basidia (Marr and Stuntz 1973).

**Figure 8.**
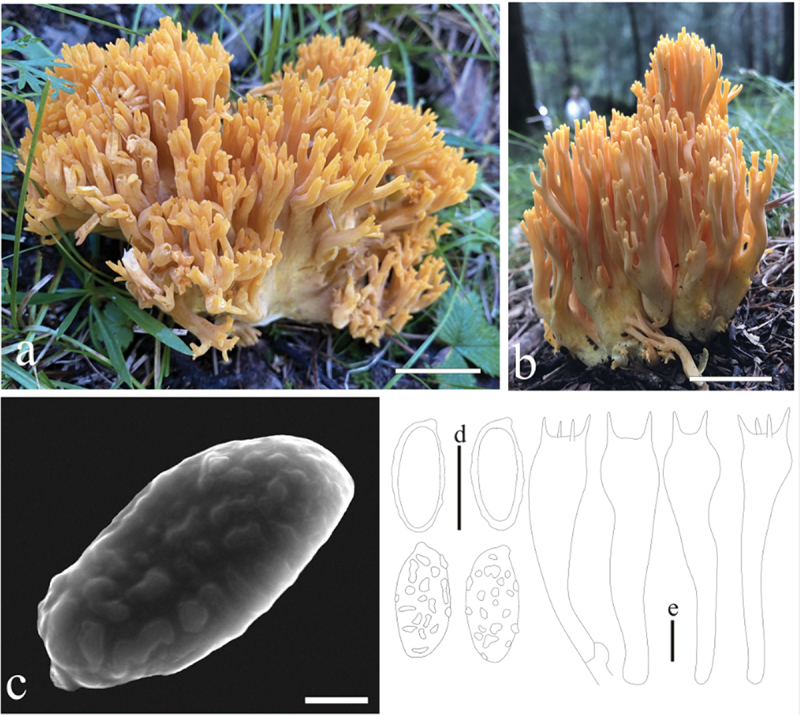
*Ramaria cyanophila*. (a – b) Basidiomata. (c) Scanning electron microscope (SEM) photographs of basidiospores. (d) Basidiospores. (e) Basidia. Scale bars: a – b = 2 cm, c = 2 μm, d – e = 10 μm.

***Ramaria*** ***cyanophila*** L. Fan, Y. Li & N. Mao, **sp. nov**. Figure 8

MycoBank: MB853046.

Etymology: *cyanophila*, referring to the cyanophilous basidiospores.

Typification: China, Shanxi Province, Guandi Mountains, Pangquangou, on the ground in coniferous forest dominated by *Picea* spp., 1,980 m elev., 25 August 2022, J.C. Lv, LJC373 (holotype BJTC FM2557). GenBank: ITS = PP537933; nrLSU = PP544908.

Basidiomata solitary, 7 cm high, 10.7 cm broad, repeatedly branched dichotomously in 3–5 ranks, coralloid, usually light yellow (#f3edac) to light orange (#ffa500); Stipe massive, fasciculate, smooth upward, pale yellow (#f3edac) to white (#ffffff), tapering gradually downward into a tangle of white mycelia; Context white (#ffffff), solid, brittle when dry, negative when exposed to ferric sulphate; Branches stout, ascending, more or less terete, more or less rugulose, polychotomous, cream (#fffdd0) below, upward pale yellow (#f3edac); Axils more and less rounded; Apices finger-like, pale orange (#ffa500), rather crowded; Taste and odour not recorded.

Hyphal system monomitic, generative hyphae simple-septate, branched, walls smooth and hyaline; Stipe tramal hyphae 2.5 − 10 µm wide, hyaline, thin-walled, parallel, clamped; Gloeoplerous hyphae not observed; Tramal hyphae of upper branches 2.5 − 10 µm wide, thin-walled, loosely parallel, hyaline, clamped; Rhizomorph hyphae of basal mat 2.5 − 8 µm diam., and inflated up to 12.5 µm, thin-walled, loosely interwoven, clamped, crystals absent; Hymenium all along the basidiomata; Basidia 42.5 − 65(−75) × 10 − 15 µm, clavate, tapering from apex to base, simple-septate, clamped, multiguttulate when mature, hyaline, strongly cyanophilous, 2/4-spored, sterigmata 5 − 7.5 µm long; Basidiospores [90/3/3] (10−)12.5 − 15 × 5 − 6.2 µm, [Q = (2−)2.27 − 3, Q_m_ = 2.51 ± 0.26], ellipsoid or elongate, pale yellow under bright field, ornamented with scattered very fine warts, inamyloid, with 1-several guttulae, strongly cyanophilous.

Habitat: Solitary on the ground in broad leaved forest dominated by *Populus* spp.

Distribution: Currently only known from Shanxi Province, northern China.

Additional specimens examined: China, Shanxi Province, Lvliang City, Jiaocheng County, Pangquangou, Badaogou, on the ground in coniferous forest dominated by *Picea* spp., 2,000 m elev., 28 August 2018, J.Z. Cao, LH260 (HSA 260); ibid, J.Z. Cao, LH261 (HSA 261).

Notes: *Ramaria cyanophila* formed a strongly supported sister lineage to *R. flavicingula* and *R. luteoaurantiaca*. *Ramaria cyanophila* appears to be the allied species phylogenetically of *R. flavicingula*, similar in white context, clamped hyphae and basidia, but *R. flavicingula* differs by its single or fasciculate stipe and small basidiospores (10.1 − 11.2 × 4.3 − 5.0 µm) (Petersen and Mu 1989). The ITS sequences of *R. cyanophila* share less than 94.62% similarity with those of *R. flavicingula*. *Ramaria luteoaurantiaca* is distinguished from *R. cyanophila* by its larger basidiomata (10 − 18 cm high, 8 − 14 cm broad) and luteo-ochraceus basidiospores. The ITS sequences of *R. cyanophila* share less than 94.96% similarity with those of *R. luteoaurantiaca* (Marchetti 2020).

**Figure 9.**
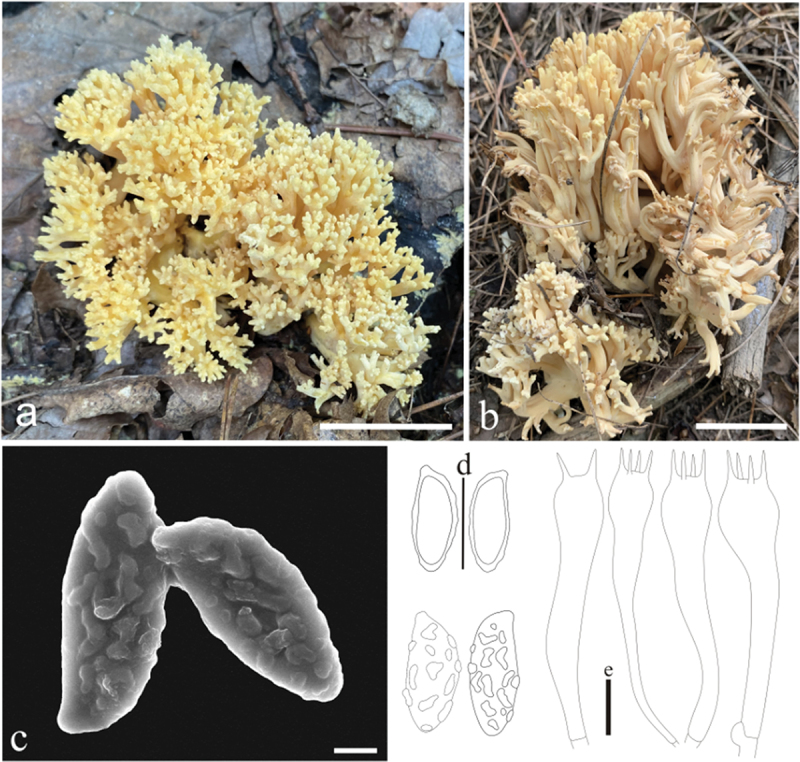
*Ramaria flavicoralloides*. (a – b) Basidiomata. (c) Scanning electron microscope (SEM) photographs of basidiospores. (d) Basidiospores. (e) Basidia. Scale bars: a – b = 2 cm, c = 2 μm, d – e = 10 μm.

***Ramaria*** ***flavicoralloides*** L. Fan, Y. Li & N. Mao, **sp. nov**. Figure 9

MycoBank: MB853048.

Etymology: *flavicoralloides*: pale yellow, coralloid, referring to the color and shape of basidiocarps.

Typification: China, Shanxi Province, Qinshui County, zhongcun forest farm, on fallen wood in a forest of *Pinus* spp., 1,658 m elev., 7 September 2023, N. Mao, MNM838 (holotype BJTC FM3520). GenBank: nrLSU = PP544902; mtSSU = PP550678; atp6 = PP556457.

Basidiomata solitary, 3 − 8 cm high, 2 − 6 cm broad, brittle, repeatedly branched dichotomously in 3 – 4 ranks, coralloid, usually obconic in outline, pale yellow (#ffe87c) to saffron-yellow (#ffd296), pale ochraceous buff (#f0ca82) on drying; Stipe 2 – 4 cm high, fasciculate, smooth upward, tapering gradually downward into a tangle of white mycelia, unchanging on drying, without any abortive stumps high on stipe. Context white (#ffffff), solid, brittle when dry, negative when exposed to ferric sulphate; Major branches 4 − 6, stout, ascending, more and less terete, saffron-yellow (#ffd296); Branches 3 − 4 ranks, polychotomous, ascending, longitudinally rugose, saffron-yellow (#ffd296), internodes diminishing gradually upward; Axils more or less rounded; Apices 1 − 2 mm long, blunt, saffron-yellow (#ffd296), ochre-brown (#f0ca82) on drying; Taste and odour not recorded.

Hyphal system monomitic, generative hyphae simple-septate, branched, walls smooth and hyaline; Stipe tramal hyphae 2 − 10 µm wide, thin-walled, parallel, hyaline, clamped; Gloeoplerous hyphae not observed; Tramal hyphae of upper branches 2.5 − 10 µm wide, thin-walled, loosely parallel, hyaline, clamped; Rhizomorph hyphae of basal mat 2 − 7 µm diam., thin-walled, loosely interwoven, clamped, without crystals; Hymenium all along the basidiomata; Basidia (36−)43 − 63(−67) × 7 − 11 µm, clavate, simple-septate, clamped, multiguttulate, strongly cyanophilous, 2/4-spored, sterigmata 4 − 7 µm long. Basidiospores [120/4/4] 7 − 10 × 4 − 5 µm [Q = (1.4−)1.75 − 2.5, Q_m_ = 2.12 ± 0.46], ellipsoid or elongate, pale yellow under bright field, slightly thick-walled, ornamented with scattered small warts, warts very low, irregular-shaped to short ridge, inamyloid, with 1-several guttulae, moderately cyanophilous.

Habitat: Solitary on the ground in coniferous and broadleaved mixed forest dominated by *Quercus* spp. and *Pinus* spp.

Distribution: Currently only known from Shanxi Province, northern China.

Additional specimens examined: China, Shanxi Province, Qinshui County, zhongcun forest farm, on fallen wood in a mixed forest of *Quercus* spp. and *Pinus* spp., 1,680 m elev., 24 August 2020, J.Z. Cao, LH1106 (BJTC FM0970); Qinshui County, Shangwoquan Village, on the ground in mixed forest dominated by *Quercus* spp., 1,620 m elev., 10 August 2022, J.C. Lv, LJC178 (BJTC FM2215); Changzhi City, Qingyuan County, Lingkongshan Mountains, on the ground in a forest of *Quercus* spp., 13 Sep. 2022, J.Z. Cao, LH1778 (BJTC FM2790).

Notes: *Ramaria flavicoralloides* is characterized by its pale yellow to saffron-yellow basidiomata, clamped hyphae, and basidia. It is morphologically and phylogenetically closely related to *Ramaria platyrugosa* having pale yellow basidiomata. However, *R. platyrugosa* has large-sized basidiomata (up to 10.5 cm high and 9 cm broad), flat and rugulose branches, and clampless hyphae and basidia.

**Figure 10.**
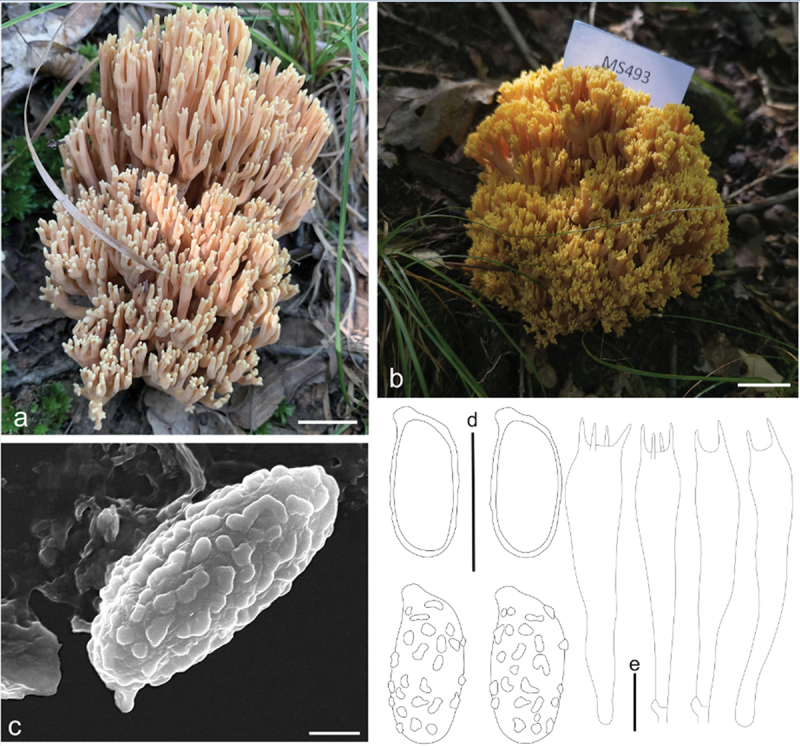
*Ramaria formosoides*. (a – b) Basidiomata. (c) Scanning electron microscope (SEM) photographs of basidiospores. (d) Basidiospores. (e) Basidia. Scale bars: a – b = 2 cm, c = 2 μm, d – e = 10 μm.

***Ramaria*** ***formosoides*** L. Fan, Y. Li & N. Mao, **sp. nov**. Figure 10

MycoBank: MB853049.

Etymology: *formosoides*, referring to its close relationship to *Ramaria formosa* in phylogeny and morphology.

Typification: China, Shanxi Province, Linfen City, Pu Village, Wulu Mountains, on the ground in broadleaf forest dominated by *Quercus* spp., 1,540 m elev., 14 August 2022, J.C. Lv, LJC280 (holotype BJTC FM2403). GenBank: ITS = PP537938; nrLSU = PP544914.

Basidiomata solitary, 8 − 10 cm high, 9.4 − 13 cm broad, repeatedly branched dichotomously in 3 – 4 ranks, coralloid, usually pale ochraceous (#dda553), brown (#dd9a53) on drying; Stipe massive, 2.5 − 4 cm high, falsely fasciculate, smooth upward, pale ochraceous (#dda553) when fresh, brown (#dd9a53) after drying, tapering gradually downward into a tangle of white mycelia, unchanging on drying; Context white (#ffffff), solid, brittle when dry, negative when exposed to ferric sulphate; Major branches stout, ascending, more or less terete, pale brown (#dda553); Branches 3 − 4 ranks, ascending, polychotomous, internodes diminishing gradually upward, pale brown (#dda553); Axils more or less rounded; Apices acute, yellow orange at first, then changing to pale ochraceous (#eab86c); Taste and odour reactions not recorded.

Hyphal system monomitic, generative hyphae simple-septate, branched, walls smooth and hyaline; Stipe tramal hyphae 2 − 6 µm wide, thin-walled, parallel, hyaline, clamped; Gloeoplerous hyphae not observed; Tramal hyphae of upper branches 2 − 7 µm wide, thin-walled, loosely parallel, hyaline, clamped; Rhizomorph hyphae of basal mat 3 − 7 µm diam., thin-walled, parallel, clamped, crystals absent; Hymenium all along the basidiomata; Basidia 42 − 65 × 8 − 10 µm, hyaline, clavate, simple-septate, clamped, multiguttulate when mature, strongly cyanophilous, 2/4-spored, sterigmata 5 − 7 µm long; Basidiospores [180/6/6] (7.5−)9 − 13(−14) × (4−)5 − 6 µm, [Q = (1.5−)1.83 − 2.75(−3.25), Q_m_ = 2.29 ± 0.38], elongate pip-shaped, pale yellow under bright field, ornamented with distinct warts, warts scattered to arranged more or less longitudinally, occasionally converging into short ridges, inamyloid, strongly cyanophilous.

Habitat: Solitary on the ground in broadleaf forest dominated by *Quercus* spp.

Distribution: Currently only known from Shanxi Province, northern China.

Additional specimens examined: China, Shanxi Province, Linfen City, Pu Village, Wulu Mountains, on the ground in broadleaf forest dominated by *Quercus* spp., 1,540 m elev., 14 August 2022, J.C. Lv, LJC287 (BJTC FM2410); Lingchuan County, Duohuo Village, on the ground in broadleaf forest dominated by *Quercus* spp., 1,182 m elev., 28 August 2023, H.Y. Fu, MS493 (BJTC FM3437); ibid. H.Y. Fu, MS504 (BJTC FM3448); ibid. 5 September 2023, N. Mao, MNM889 (BJTC FM3569); ibid. N. Mao, MNM893 (BJTC FM3573).

Notes: *Ramaria formosoides* is recognized by its massive and falsely fasciculate stipe, clamped hyphae, and spore ornamentation. *Ramaria formosa* formed a strongly supported sister lineage to *R. formosoides* (Figures 1 and 2) and shares similar branching pattern, clamped basidia, and tramal hyphae. However, *R. formosa* is distinguished *R. formosoides* by its larger basidiomata (15 × 9 cm) and pale orange context that is positive when exposed to ferric sulphate (Quélet 1888). DNA analysis revealed that the ITS sequences of *R. formosa* share less than 82.45% similarity with those of *R.* *formosoides*.

**Figure 11.**
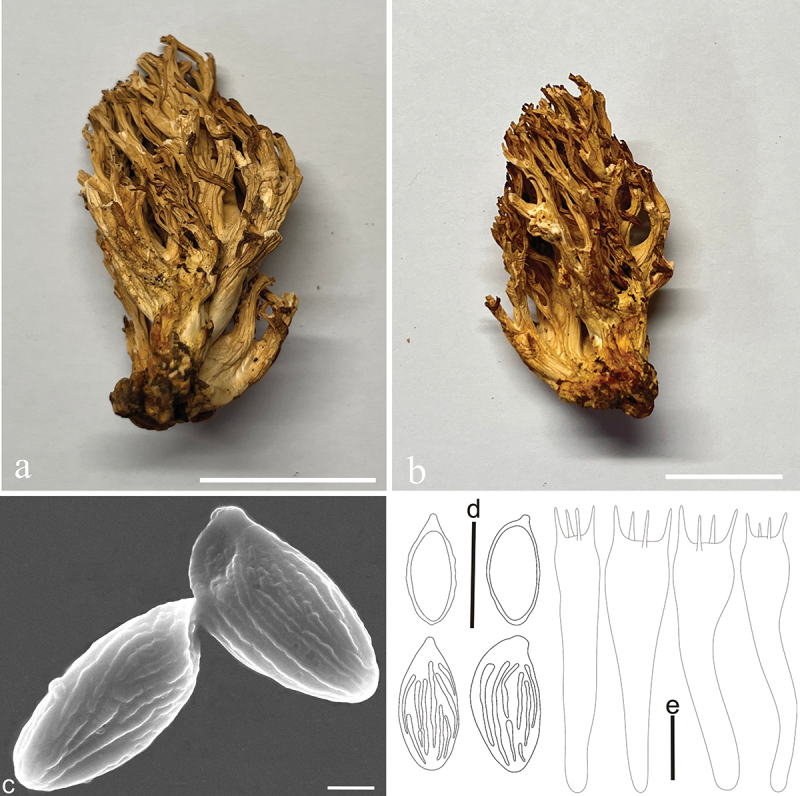
*Ramaria lingkongshanensis*. (a – b) Basidiomata. (c) Scanning electron microscope (SEM) photographs of basidiospores. (d) Basidiospores. (e) Basidia. Scale bars: a – b = 2 cm, c = 2 μm, d – e = 10 μm.

***Ramaria*** ***lingkongshanensis*** L. Fan, Y. Li & N. Mao, **sp. nov**. Figure 11

MycoBank: MB853051.

Etymology: *lingkongshanensis*: referring to type locality.

Typification: China, Shanxi Province, Changzhi City, Qinyuan County, Lingkong Mountains, Shengshoumiao, on the ground in mixed forest, 1,520 m elev., 24 July 2021, J.C. Lv, LJC013 (holotype BJTC FM1723). GenBank: ITS = PP537934; nrLSU = 544909.

Basidiomata solitary, 6 cm high, 3 cm broad, repeatedly branched dichotomously in 5 – 6 ranks, coralloid, usually pale ochraceous (#d6a876) on drying; Stipe 1 cm high, compound to fasciculate in groups of 2, smooth upward, pale ochraceous (#d6a876) after drying, tapering gradually downward into a tangle of white mycelia; Context white (#ffffff), brittle when dry, negative when exposed to ferric sulphate; Major branches 3 − 4, terete, more or less rugulose, pale ochraceous (#d6a876); Branches 5 − 6 ranks, polychotomous, internodes diminishing gradually upward, pale ochraceous (#d6a876); Apices acute, pale ochraceous (#d6a876) to ochraceous (#ca8f4f); Taste and odour not recorded.

Hyphal system monomitic, generative hyphae simple-septate, branched, walls smooth and hyaline; Stipe tramal hyphae 3 − 7 µm wide, thin-walled, interwoven, hyaline, clamped; Gloeoplerous hyphae not observed; Tramal hyphae of upper branches 2 − 5 µm wide, thin-walled, loosely parallel, hyaline, clamped; Rhizomorph hyphae of basal mat 3 − 7 µm diam., thin-walled, interwoven, hyaline, clamped, crystals absent; Hymenium all along the basidiomata; Basidia 50 − 65(−70) × 7 − 10(−12) µm, clavate, simple-septate, clampless, multiguttulate when mature, strongly cyanophilous, 4-spored, sterigmata 3.5 − 7 µm long; Basidiospores [60/2/2] 11 − 13(−15) × 4.5 − 5 µm, [Q = (2.2−)2.4 − 2.7, Q_m_ = 2.53 ± 0.16] ellipsoid, pale yellow under bright field, ornamented with numerous, elongated ridges arranged longitudinally, inamyloid, strongly cyanophilous.

Habitat: Solitary on the ground in broadleaf forest dominated by *Quercus* spp.

Distribution: Currently only known from Shanxi Province, northern China.

Additional specimen examined: China, Shanxi Province, Changzhi City, Qinyuan County, Lingkong Mountains, Shengshoumiao, on the ground in mixed forest, 1,520 m elev., 24 July 2021, J.C. Lv, LJC014 (BJTC FM1724).

Notes: *Ramaria lingkongshanensis* is characterized by clamped hyphae, clampless basidia, and inamyloid basidiospores. *Ramaria lingkongshanensis* is morphologically and phylogenetically closely related to *Ramaria pallidissima* schild & G. Ricci, but the ITS sequences of *R. pallidissima* share less than 92.49% similarity with those of *R. lingkongshanensis* (Franchi and Marchetti 2022). *Ramaria lingkongshanensis* is usually collected for eating by locals.

**Figure 12.**
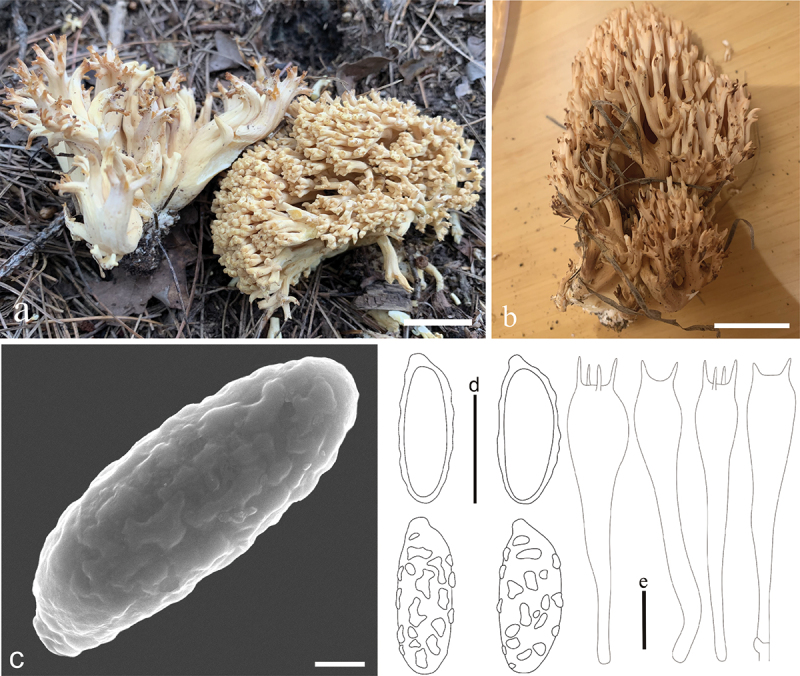
*Ramaria obtusa*. (a – b) Basidiomata. (c) Scanning electron microscope (SEM) photographs of basidiospores. (d) Basidiospores. (e) Basidia. Scale bars: a – b = 2 cm, c = 2 μm, d – e = 10 μm.

***Ramaria obtusa*** L. Fan, Y. Li & N. Mao, **sp. nov**. Figure 12

MycoBank: MB853053.

Etymology: *obtusa*, obtuse, referring to the shape of branch-tip of basidiomata.

Typification: China, Shanxi Province, Jincheng City, Qinshui County, zhongcun forest farm, on the ground in mixed forest dominated by *Quercus* spp., 1,680 m elev., 7 September 2023, N. Mao, MNM839 (BJTC FM3521). GenBank: ITS = PP537945; nrLSU = PP544922.

Basidioma solitary, 8.8 − 17 cm high, 4.8 − 9 cm broad, repeatedly branched dichotomously in 3 – 5 ranks, coralloid, usually pale brownish-yellow (#d9a36c); Stipe up to 1.6 − 3 cm high, fasciculate, tapering gradually downward into a tangle of white mycelia; Context white (#ffffff), brittle when dry, negative when exposed to ferric sulphate, unchanging on drying; Major branches 2 − 4, ascending, terete, pale brown (#d9a36c); Branches 3 − 5 ranks, ascending, polychotomous, internodes diminishing gradually upward, pale brown (#d9a36c); Apices obtuse, pale brown (#d9a36c); Taste and odour reactions not recorded.

Hyphal system monomitic, generative hyphae simple-septate, branched, walls smooth and hyaline; Stipe tramal hyphae 5 − 10 µm wide, and inflated up to 15 µm, thin-walled, interwoven, hyaline, clamped; Gloeoplerous hyphae not observed; Tramal hyphae of upper branches 2.5 − 12.5 µm wide, thin-walled, loosely parallel, hyaline, clamped; Rhizomorph hyphae of basal mat 2.5 − 5 µm diam., thin-walled, clamped, crystals absent; Hymenium all along the basidiomata; Basidia 52.5 − 77.5 × 7.5 − 10.5 µm, clavate, simple-septate, clamped, multiguttulate when mature, strongly cyanophilous, 2/4-spored, sterigmata 2.5 − 7.5 µm long; Basidiospores [60/2/2]10 − 12.5(−15) × 4.5 − 5 µm, [Q = 2 − 3(−3.3), Q_m_ = 2.45 ± 0.38], ellipsoidal, pale yellow under bright field, ornamented with irregular-shaped warts, warts very low, discrete, more or less arranged longitudinally, inamyloid, with 1-several guttulae, strongly cyanophilous.

Habitat: Solitary on the ground in broadleaf forest dominated by *Quercus* spp.

Distribution: Currently only known from Shanxi Province, northern China.

Additional specimen examined: China, Shanxi Province, Jincheng City, Qinshui County, zhongcun forest farm, on the ground in mixed forest dominated by *Quercus* spp., 1,680 m elev., 7 September 2023, N. Mao, MNM836 (BJTC FM3519).

Notes: Phylogenetic analyses showed that *R. obtusa* forms an independent clade, sister to *Ramaria flavescens* or *Ramaria flavescentoides* (Figures 1 and 2). However, *R. flavescens* has a higher and single stipe (5 cm vs. 1.6 − 3 cm high), white or cream branches, and pinkish salmon context that is positive when exposed to ferric sulphate (Petersen 1974); *Ramaria flavescentoides* is distinguished by its pale orange apices with light greenish grey tint and stellate crystalline material in the hyphae of upper branches (Hanif et al. 2019). *Ramaria obtusa* shares less than 90.0% similarity in ITS sequence with that of *R. flavescens*, and less than 89.94% with *R.* *flavescentoides*.

**Figure 13.**
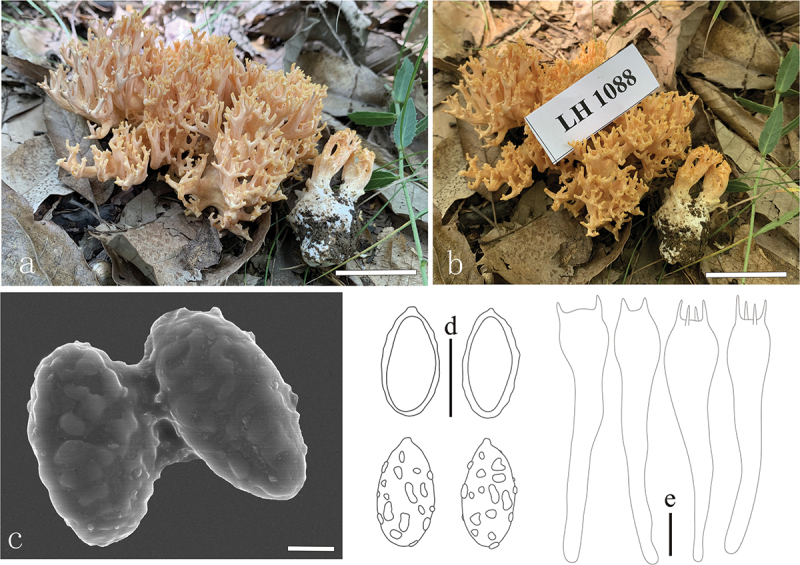
*Ramaria persicinoflava*. (a – b) Basidiomata. (c) Scanning electron microscope (SEM) photographs of basidiospores. (d) Basidiospores. (e) Basidia. Scale bars: a – b = 2 cm, c = 2 μm, d – e = 10 μm.

***Ramaria*** ***persicinoflava*** L. Fan, Y. Li & N. Mao, **sp. nov**. Figure 13

MycoBank: MB853055.

Etymology: *persicinoflava*, referring to the color of basidiocarp.

Typification: China, Shanxi Province, Qinshui County, Shangwoquan Village, 112°3’5’’E, 35°36’57’’N, elev. 1,170 m, 25 August 2020, collected by H. Liu LH1088 (BJTC FM1070). GenBank: ITS = PP537923; nrLSU = PP544896; atp6 = 556453.

Basidiomata solitary, 8 cm high, 6 cm broad, brittle, repeatedly branched dichotomously in 3 – 5 ranks, coralloid, usually slightly yellow (#ffd581) to white (#ffffff) with a little pale flesh-pink (#fff3f5), becoming brownish (#e0b586) in age; Stipe massive, 1.6 cm high, fasciculate, smooth upward, pale yellow (#ffd581) to white (#ffffff), pale ochraceous buff (#e0b586) on drying, tapering gradually downward into a tangle of white mycelia, without abortive branches; Context white (#ffffff), solid, brittle when dry, negative when exposed to ferric sulphate; Branches 3 − 5 ranks, polychotomous, ascending, internodes gradually wide, terete, more or less rugulose, pale yellow (#ffd581); Axils more or less rounded; Apices subacute, buff-yellow (#ffcb81); Taste and odour not recorded.

Hyphal system monomitic, generative hyphae simple-septate, branched, walls smooth and hyaline; Stipe tramal hyphae 2.5 − 7.5 µm wide, and inflated up to 12.5 µm, thin-walled, parallel, hyaline, clampless; Gloeoplerous hyphae not observed; Tramal hyphae of upper branches 2.5 − 7.5 µm wide, and inflated up to 10 µm, thin-walled, loosely parallel, hyaline, clampless; Rhizomorph hyphae of basal mat 2.5 − 5 µm diam., and inflated up to 10 µm, thin-walled, loosely interwoven, clampless, crystals absent; Hymenium all along the basidiomata; Basidia (50−) 55 − 67.5 × 10 − 12.5 µm, clavate, simple-septate, clampless, multiguttulate, cyanophilous, 2/4-spored, sterigmata 2.5 − 5 µm long; Basidiospores [30/1/1] (10.2−) 11.2 − 15 × 4.5 − 6.25 µm [Q = (2.2−) 2.27 − 2.72 (−3), Q_m_ = 2.48 ± 0.25], elongate-ellipsoid, lightly yellow under bright field, ornamented with scattered small warts, warts very low, nearly round, elliptical, and irregularly short ridged, inamyloid, apiculus not conspicuous, with 1-several guttulae, moderately cyanophilous.

Habitat: Solitary or scattered on the ground in broadleaf forest dominated by *Quercus* spp.

Distribution: Currently only known from Shanxi Province, northern China.

Notes: *Ramaria persicinoflava* is recognized by its pinkish orange color, wide internodes and axils, and spore ornamentation. *Ramaria persicinoflava* formed a strongly supported sister lineage to *R. subcolumnaris*, and both resemble in branching pattern, clampless hyphae, also sharing a similar basidiomata and spore size and shape. *Ramaria subcolumnaris* differs from the phylogenetically closest related species *R. persicinoflava* by its single stipe, subcolumn outline, and buff-yellow to white basidiomata that lack pinkish orange. Moreover, DNA analysis revealed that the ITS sequence of *R. persicinoflava* shares less than 96.7% similarity with those of *R.* *subcolumnaris*.

**Figure 14.**
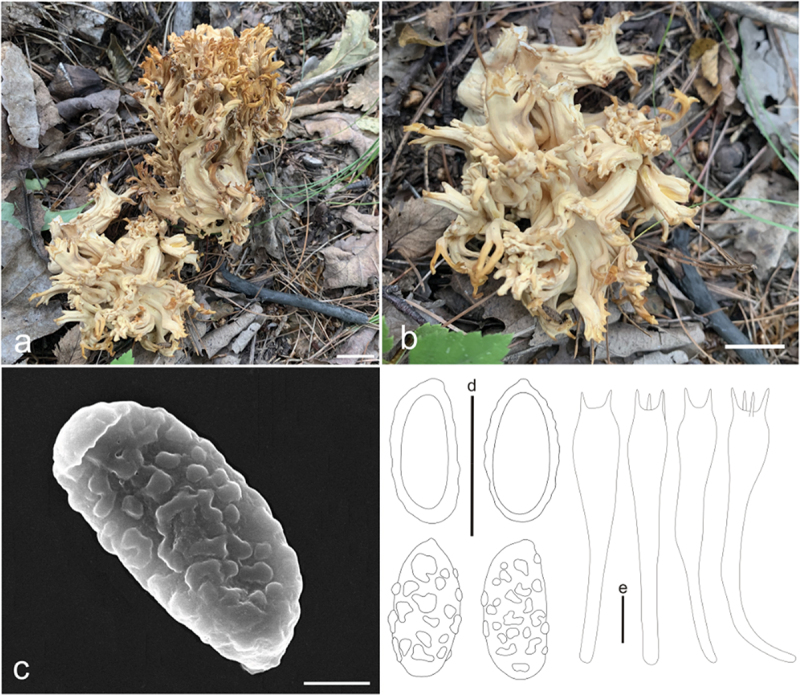
*Ramaria platyrugosa*. (a – b) Basidiomata. (c) Scanning electron microscope (SEM) photographs of basidiospores. (d) Basidiospores. (e) Basidia. Scale bars: a – b = 2 cm, c = 2 μm, d – e = 10 μm.

***Ramaria platyrugosa*** L. Fan, Y. Li & N. Mao, **sp. nov**. Figure 14

MycoBank: MB853056.

Etymology: *platyrugosa*, refers to the flat and rugulose branches of basidiocarps.

Typification: China, Shanxi Province, Jincheng City, Qinshui County, zhongcun forest farm, on the ground in mixed forest dominated by *Quercus* spp., 1,680 m elev., 7 September 2023, N. Mao, MNM844 (BJTC FM3526). GenBank: ITS = PP537925; nrLSU = PP544898; mtSSU = PP550674.

Basidiomata solitary, 10.5 cm high, 9 cm broad, repeatedly branched dichotomously in 4 – 5 ranks, coralloid, usually pale yellow (#fff7dc) to yellow (#ffe4a9), brownish-yellow (#d68900) on drying; Stipe up to 2 − 3 cm high, compound to fasciculate in groups of 2 – 3, smooth upward, tapering gradually downward into a tangle of white mycelia; Context white (#ffffff), solid, brittle when dry, negative when exposed to ferric sulphate; Major branches 2 − 3, flat and rugulose, pale yellow (#fff7dc) below; Branches 4 − 5 ranks, polychotomous, longitudinally rugose, pale yellow (#ffe4a9); Apices 2 − 3 mm long, obtuse, pale brown (#dd8d00); Taste and odour reactions not recorded.

Hyphal system monomitic, generative hyphae simple-septate, branched, walls smooth and hyaline; Stipe tramal hyphae 2.5 − 7.5 µm wide, thin-walled, hyaline, clampless; Gloeoplerous hyphae not observed; Tramal hyphae of upper branches 5 − 10 µm wide, thin-walled, hyaline, clampless; Rhizomorph hyphae of basal mat 2.5 − 7.5 µm diam., thin-walled, hyaline, clampless, without crystals; Hymenium all along the basidiomata; Basidia 46 − 62.5 × 7.5 − 11.3 µm, clavate, simple-septate, clampless, multiguttulate when mature, strongly cyanophilous, 2/4-spored, sterigmata 3.8 − 7.5 µm long; Basidiospores [30/1/1] 10 − 12.5 × 3.8 − 5 µm, [Q = 2 − 2.67, Q_m_ = 2.46 ± 0.34], ellipsoidal, pale yellow under bright field, ornamented with distinct, nearly round or irregular-shaped warts, inamyloid, apiculate, strongly cyanophilous.

Habitat: Solitary on the ground in broadleaf forest dominated by *Quercus* spp.

Distribution: Currently only known from Shanxi Province, northern China.

Note: *Ramaria platyrugosa* is recognized by its flat and rugulose branches, clampless hyphae, and spores ornamented with warts. *Ramaria platyrugosa* formed a strongly supported sister lineage to *R. apicaliochracea*, a new species described in this study, and both resemble clampless hyphae and basidia, spores size and shape. However, *R. apicaliochracea* is distinguished from *R. platyrugosa* by its smooth and terete branches and cream to pale yellow stipe. The nrLSU sequences of *R. apicaliochracea* share less than 98.7% similarity with those of *R.* *platyrugosa*.

**Figure 15.**
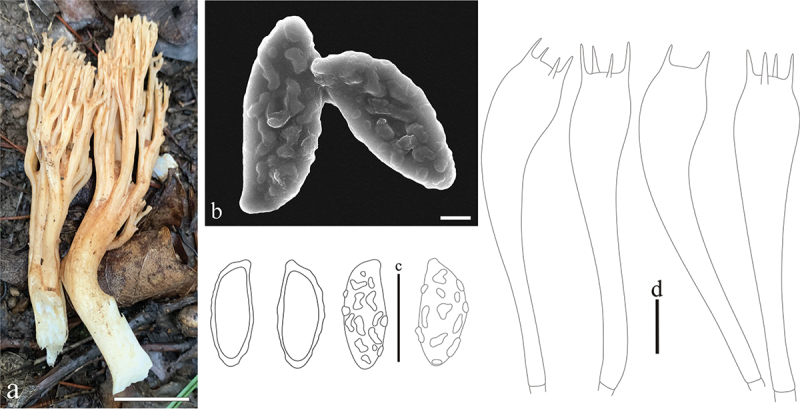
*Ramaria subcolumnaris*. (a) Basidiomata. (b) Scanning electron microscope (SEM) photographs of basidiospores. (c) Basidiospores. (d) Basidia. Scale bars: a = 2 cm, b = 2 μm, c – d = 10 μm.

***Ramaria subcolumnaris*** L. Fan, Y. Li & N. Mao, **sp. nov**. Figure 15

MycoBank: MB853057.

Etymology: *subcolumnaris*, sub-column, referring to the shape of basidiomata in profile.

Typification: China, Shanxi Province, Linfen City, Pu County, Wulu Mountain, on the ground in broadleaf forest dominated by *Quercus* spp., 1,735.6 m elev., 9 August 2022, N. Mao, MNM552 (holotype BJTC FM2198). GenBank: ITS = PP537930; nrLSU = PP544905; atp6 = PP556460.

Basidiomata solitary, 8.5 − 10.3 cm high, 1.8 − 2.1 cm broad, brittle, repeatedly branched dichotomously in 4 – 5 ranks, coralloid, usually sub-columnar in profile, pale buff-yellow (#ffd297) to white (#ffffff), light brown (#db8e00) on drying; Stipe up to 4.2 − 5 cm high, pale brown (#eaad6f), tapering gradually downward into a tangle of white mycelia; Context white (#ffffff), solid, brittle when dry, negative when exposed to ferric sulphate; Major branches 4 − 5, ascending straightly, more or less terete, with smooth walls, buff-yellow (#ffd297); Branches 4 − 5 ranks, polychotomous ascending straightly, light brown (#ffd297), internodes diminishing gradually upwards; Axils more or less rounded; Apices obtuse, rather crowded, light brown (#ffd297); Taste and odour not recorded.

Hyphal system monomitic, generative hyphae simple-septate, branched, walls smooth and hyaline; Stipe tramal hyphae of stipe 2 − 8 µm wide, and inflated up to 11 µm, thin-walled, loosely interwoven, hyaline, clampless; Gloeoplerous hyphae not observed; Tramal hyphae of upper branches 2 − 9 µm wide, thin-walled, loosely parallel, hyphae, clampless; Rhizomorph hyphae of basal mat 2 − 7 µm diam., and inflated up to 11 µm, thin-walled, loosely interwoven, clampless, crystals absent; Hymenium all along the basidiomata; Basidia (42.5−)50 − 75(−80) × 10 − 12.5(−15) µm, clavate, hyaline, simple-septate, clampless, multiguttulate, strongly cyanophilous, 2/4-spored, sterigmata 2.5 − 5 µm long; Basidiospores [90/3/3] (10−)11 − 14 × 4 − 5 µm, [Q = (2−)2.4 − 3.25(−3.5), Q_m_ = 2.78 ± 0.38], elongate pip-shaped, light yellow under bright field, ornamented with distinct, discrete warts, warts irregular-shaped to short-ridged inamyloid, apiculate, with 1-several guttulae, moderately cyanophilous.

Ecology and habitat: Solitary on the ground in broadleaf forest dominated by *Quercus* spp., currently only known from Shanxi Province, northern China.

Additional specimens examined: China, Shanxi Province, Wutai Mountain, Zhijiayu Village, on the ground in the forest, WT001 (BJTC FM1977); Linfen City, Pu County, Wulu Mountain, on the ground in broadleaf forest dominated by *Quercus* spp., 1,644.6 m elev., 26 August 2022, N. Mao, MNM793 (BJTC FM2607).

Notes: *Ramaria subcolumnaris* is recognized by its subcolumn profile, straightly ascending branches, inamyloid context, and clampless hyphae. The new species formed a strongly supported sister lineage to *R. persicinoflava*, and both resemble in branching pattern, clampless hyphae, also sharing similar basidiomata and spore size and shape. However, *R. persicinoflava* morphologically differs from *R. subcolumnaris* by its fasciculate stipe and wide internodes and axils. Moreover, DNA analysis revealed that the ITS sequences of *R. subcolumnaris* share less than 96.7% similarity with those of *R. subcolumnaris*.

## 4. Discussion

There are seven species of *Ramaria* reported from Shanxi Province before this study, but all were determined only based on morphology, lacking molecular data, which are documented as follows: *R. apiculata* (Fr.) Donk (Pan 2018), *R. bourdotiana* Maire (Li and Liu 1988; Pan 2018), *R. ephemeroderma* R.H. Petersen & M. Zang (Pan 2018); *R. flaccida* (Fr.) Bourdot [= *Phaeoclavulina flaccida* (Fr.) Giachini] (Liu 1985, 1991); *R. flava* (Schaeff.) Quél (Du et al. 1979; Li 1985; Li and Liu 1988; Liu et al. 1991; Pan 2018); *R. invalii* (Cotton and Wakef.) Donk [= *Phaeoclavulina eumorpha* (P. Karst.) Giachini]; *R. stricta* (Pers.) Quél (Du et al. 1979; Li and Liu 1988; Liu et al. 1991; Yang et al. 1992; Zhu et al. 1992; Wang et al. 2008). Unfortunately, no species mentioned above was confirmed by the present study as the specimens cited in these previous studies are not available. Of the seven species, two species have been transferred to the genus *Phaeoclavulina*, i.e. *R. flaccida* (= *Phaeoclavulina flaccida*), *R. invalii* (= *Phaeoclavulina eumorpha*). The remaining five species, morphologically, *R. ephemeroderma* is similar to *R. affinis* in branching pattern of basidiomata and stipe compound to fasciculate in groups of 2 – 3, but it is differentiated by its humicolous habit in broadleaf forests dominated by *Populus* spp. and smaller basidiomata (4 − 7 cm high *vs*. 10 − 13 cm high); *R. flava* is similar to *R. cyanophila* in light yellow basidiomata and warty basidiospores, but *R. flava* has bruising red and blood-red basidiomata; *R. stricta* is similar to *R. formosoides* in basidiomatal colour and size, but *R. stricta* has smooth basidiospores and basidiomata bruising brown and red-brown upon collecting; *R. bourdotiana* and *R. apiculate* are completely different in appearance of basidiomata. The basidiomata of *R. bourdotiana* are dendritic in the whole cluster, having very thin and dense branches and serrated apices. The basidiomata of *R. apiculate* is gregarious in caespitose clusters, having yellowish-brown, wide, and flat branches.

The present multilocus phylogenetic analysis and morphological data suggest that there are at least 13 *Ramaria* species in Shanxi Province including 12 new species and a new report to China. Most of them limited their distribution in the south region of Shanxi Province and are associated with oaks, only *Ramaria cyanophila*, *R. conferta*, and *R. affinis* were distributed in north area where it is associated with *Picea* spp. in subalpine region. This geographic pattern implied that both the climate and host tree may significantly influence the distribution of *Ramaria* species.

**Key to the species of *Ramaria* from Shanxi Province, northern China**

1. Basidiomata lignicolous or humicolous.......2

1. Basidiomata terricolous.....4

2. Basidiomata bruising brown and red-brown upon collecting............ *R. stricta*

2. Basidiomata not bruising......3

3. Basidiomata dendritic in whole cluster, branches very thin and dense, apices serrated.......*R. bourdotiana*

3. Basidiomata coralloid, sparingly branched, apices acute......*R. ephemeroderma*

4. Basidiomata small in size (<10 cm height and wide of the entire basidiomata).....5

4. Basidiomata usually large in size (>10 cm height or wide of the entire basidiomata).....9

5. Apices acute......6

5. Apices obtuse or finger-like..........7

6. Terrestrial in coniferous forests dominated by *Picea* spp. and *Pinus* spp.; Basidiomata gregarious in caespitose clusters, branches wide and flat......*R. apiculate*

6. Terrestrial in coniferous and broad-leaved mixed forest dominated by *Quercus* spp. and *Pinus* spp.; Basidiomata solitary, and branches terete................*R. lingkongshanensis*

7. Branches dispersing, internodes gradually wide............................................................................*R. persicinoflava*

7. Branches ascending, more and less terete, internodes diminishing gradually upward.......8

8. Basidia clamped.......*R. flavicoralloides*

8. Basidia clampless................................................*R. conferta*

9. Basidiospores smooth......10

9. Basidiospores ornamented with scattered warts or ridges...........................................................................................11

10. Basidiomata subcolumn in outline, relatively narrow (4.5 − 5 cm broad)......*R. affinis*

10. Basidiomata obconic in outline, relatively wide (up to 11.5 cm broad)................................*R. cadmioaurantiaca*

11. Branches flat and rugulose......12

11. Branches more or less terete, with smooth walls......14

12. Basidiomata bruising red and blood-red upon collecting......*R. flava*

12. Basidiomata not bruising........13

13. Tramal hyphae parallel, clamped.....*R. barenthalensis*

13. Tramal hyphae parallel, clampless........*R. platyrugosa*

14. Basidiomata light orange........................*R. cyanophila*

14. Basidiomata saffron-yellow to light brown...........15

15. Tramal hyphae clamped................................................16

15. Tramal hyphae clampless........17

16. Branches bend in the same direction, warts on basidiospore surface very low, irregular-shaped........*R. obtusa*

16. Branches ascending, more and less terete and straight, warts on basidiospore surface very distinct, occasionally converging into short ridges................................*R. formosoides*

17. Tramal hyphae loosely interwoven, inflated up to 11 µm........*R. subcolumnaris*

17. Tramal hyphae parallel, not conspicuously inflated........*R.apicaliochracea*
